# Population genomics identifies patterns of genetic diversity and selection in chicken

**DOI:** 10.1186/s12864-019-5622-4

**Published:** 2019-04-02

**Authors:** Diyan Li, Yan Li, Miao Li, Tiandong Che, Shilin Tian, Binlong Chen, Xuming Zhou, Guolong Zhang, Uma Gaur, Majing Luo, Kai Tian, Mengnan He, Shen He, Zhongxian Xu, Long Jin, Qianzi Tang, Yifei Dai, Huailiang Xu, Yaodong Hu, Xiaoling Zhao, Huadong Yin, Yan Wang, Rongjia Zhou, Chaowu Yang, Huarui Du, Xiaosong Jiang, Qing Zhu, Mingzhou Li

**Affiliations:** 1 Institute of Animal Genetics and Breeding, College of Animal Science and Technology, Sichuan Agricultural University, Chengdu, China; 2 Novogene Bioinformatics Institute, Beijing, China; 3 Division of Genetics, Department of Medicine, Brigham and Women’s Hospital, Harvard Medical School, Boston, USA; 4 Department of Animal Science, Oklahoma State University, Stillwater, OK USA; 5 Hubei Key Laboratory of Cell Homeostasis, Laboratory of Molecular and Developmental Genetics, College of Life Sciences, Wuhan University, Wuhan, China; 6 Sichuan Animal Science Academy, Chengdu, China

**Keywords:** Population genomics, Genetic diversity, Selection, Chicken

## Abstract

**Background:**

There are hundreds of phenotypically distinguishable domestic chicken breeds or lines with highly specialized traits worldwide, which provide a unique opportunity to illustrate how selection shapes patterns of genetic variation. There are many local chicken breeds in China.

**Results:**

Here, we provide a population genome landscape of genetic variations in 86 domestic chickens representing 10 phenotypically diverse breeds. Genome-wide analysis indicated that sex chromosomes have less genetic diversity and are under stronger selection than autosomes during domestication and local adaptation. We found an evidence of admixture between Tibetan chickens and other domestic population. We further identified strong signatures of selection affecting genomic regions that harbor genes underlying economic traits (typically related to feathers, skin color, growth, reproduction and aggressiveness) and local adaptation (to high altitude). By comparing the genomes of the Tibetan and lowland fowls, we identified genes associated with high-altitude adaptation in Tibetan chickens were mainly involved in energy metabolism, body size maintenance and available food sources.

**Conclusions:**

The work provides crucial insights into the distinct evolutionary scenarios occurring under artificial selection for agricultural production and under natural selection for success at high altitudes in chicken. Several genes were identified as candidates for chicken economic traits and other phenotypic traits.

**Electronic supplementary material:**

The online version of this article (10.1186/s12864-019-5622-4) contains supplementary material, which is available to authorized users.

## Background

Since the domestication of the red jungle fowl (*Gallus gallus*) (approximately 8000 to 5400 BC) in Asia [1], domestic chickens (*Gallus Gallus domesticus*) have been subject to the combined effects of natural and artificial selection. This has resulted in marked genetic diversity in a number of traits, leading to highly specialized chicken lines with unique traits, such as cockfighting fowls, bantams, meat and egg producing chickens. There are hundreds of phenotypically distinguishable domestic chicken breeds or lines worldwide [2], providing a unique opportunity to trace the history of domesticated poultry and define the signatures of selection resulting from both domestication and the natural environment. For example, game fowl are a group of breeds selected specifically for cockfighting, and fighting cocks possess congenital aggression towards all males of the same species [3]. Chickens’ wild ancestors are of great importance when considering the maintenance and improvement of domestic chicken breeds through introgression of genetic variation from wild-type genomes.

The Tibetan chicken is a breed endemic to China and is mainly distributed in Qinghai Province and the Tibetan Plateau. This population exhibits many phenotypic adaptations to the alpine, low-air-pressure environment [4]. In contrast, lowland chickens have experienced very strong selection for traits of biological and agricultural importance. The comparative analysis of Tibetan and lowland chicken genomes has the potential to shed light on the genetic components that are shaped by high-altitude adaptations in the Tibetan chicken.

To further investigate genomic variations underlying the domestication of chicken breeds and the high-altitude adaptation of Tibetan chickens, the whole genomes of 86 domestic chickens, including 50 lowland chickens from 9 phenotypically diverse breeds in China and 36 Tibetan chickens from 6 Qinghai-Tibetan Plateau localities (Additional file 1: Table S1), together with 5 red jungle fowls (RJFs), were used for a comparative population genomics analysis; we reported these data in a previous study [5].

## Results and discussion

### Genetic diversity, population structure and introgressions

**Fig. 1:**
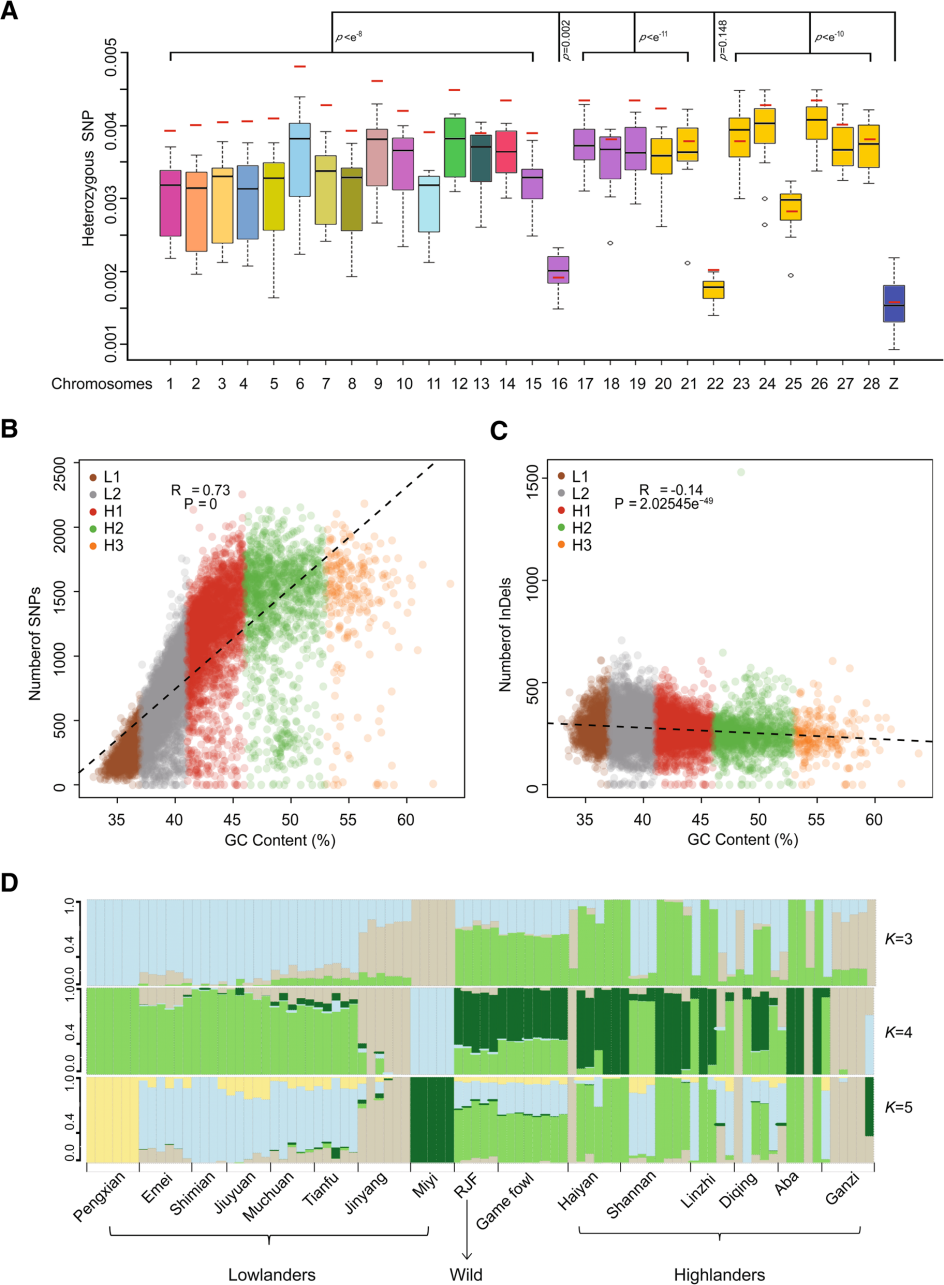
Heterozygous SNPs in each chicken chromosome; correlation between variations and GC content of the genome and population structures. **a** Boxplot showing the difference in the number of heterozygous SNP per base pair between the Z chromosome and all autosomes. The black lines inside the boxes represent the median of all 86 domestic chickens. The red lines represent the median of all five RJFs. A two-tailed *t*-test was conducted to detect significant differences between the Z chromosome and all autosomes. Correlation between the number of SNPs (**b**) or the number of indels (**c**) in 91 chickens and the GC level in the isochores of the chicken genome. **d** Population structures with the number of ancestral clusters, *K*, ranging from 3 to 5

We analyzed the genetic diversity of 91 chicken genomes and identified a total of 5.27–9.59 million SNPs for each breed (Additional file 1: Figure S1). A total of 1398–4716 specific SNPs were detected for each breed/population (Additional file 1: Table S2). A small number of heterozygous breed-specific SNPs (7–89) were found for each breed. Distribution of heterozygous SNPs within each non-overlapping 500-kb window along both sex chromosomes and autosomes indicated that the Z chromosome had far fewer heterozygous SNPs than any autosome (*p* < 0.05) except for chromosome 22 (*p* = 0.148) (Fig. 1, Additional file 1: Figure S2A). A previous study also showed that genetic variation was significantly lower at Z-linked than at autosomal loci [6]. In addition, we performed pairwise comparisons of the genome-wide variation between the 15 domestic chicken populations. Once again, we found that higher population genetic differentiation was detected in the Z chromosome than in any of the autosomes (Additional file 1: Figure S3). The genomic level of variation between sex and autosomal chromosomes possibly help to reveal the long-term history of sexual selection in a species [7].

To examine the potential impact of GC content on genetic variations, we characterized chicken genome into five isochore families defined by different GC levels, as described by numerous references [8, 9]. The chicken genomes were segmented into isochores by increasing GC levels from L1 to H3 (Additional file 1: Figure S2.B). The overall distribution of the lengths of isochores showed that more than 40% of the isochores had 37–41% GC content (Additional file 1: Figure S2.C). We used pearson correlation coefficient to calculate the relationship between GC content and number of SNPs/indels. The number of SNPs and the GC level are positively correlated in the isochores of the chicken genome (*r* = 0.73, *p* = 0) (Fig. 1b and Additional file 1: Figure S4). Surprisingly, the number of indels and the GC level in the isochores are negatively correlated in the whole chicken population (*r* = − 0.14, *p* = 0) (Fig. 1c) and each chicken breed/population (Additional file 1: Figure S5). The opposite trends of SNP and indel density in the context of GC level are probably due to crossing over event, the influence of natural selection and environmental pressure. The isochore families consist of 15.09, 40.67, 29.27, 12.41, and 2.55% of the chicken genome for L1, L2, H1, H2, and H3 isochore, respectively. The H1 isochore family contains 40.46% of SNPs and 32.90% of indels (Additional file 1: Figure S6; Table S3). Herein, we show that H1 isochore is the dominant source of genetic variation in the chicken genome. Although, isochore families H2 (20.36% SNP, 13.08% indels) and H3 (4.27% SNP, 2.74% indels) also represents remarkable variation, while present in small amounts in the genome. An analysis of SNPs in 91 fowls shows that the single nucleotide mutation rate is highly dependent on the GC content of the genome, which is also the case in humans [10].

Linkage disequilibrium (LD) analysis showed that Tibetan chicken populations had a faster LD decay rate than other domestic chicken breeds, as did RJFs (Additional file 1: Figure S7A), reflecting a relatively high level of inbreeding under artificial breeding programs and, consequently, lower genomic diversity in the more heavily domesticated chickens. To consider the possible genetic admixture among the populations, we also performed population structure analysis with a full maximum-likelihood approach using *frappe* [11], which estimates individual ancestry and admixture proportions assuming *K* ancestral populations (Fig. 1d; Additional file 1: Figure S7B). At *K* = 3, we observed a division between the wild fowls and modern breeds as well as the populations living at high and low altitudes. However, the red jungle fowl share a similar genetic background with game fowl based on K = 3, 4, 5. This indicates that red Jungle fowl interbreed with domestic chicken and believed to be a primary progenitor of game fowl. Mutual introgression was observed among chicken breeds, and the population structure also showed that the formation of phenotypically distinguishable domestic chicken breeds driven by artificial selection. For example, we found that chickens of highland such as Ganzhi, Shannan, Linzhi, and Diqing shared the genetic contribution with lowlanders at K = 5. Interestingly, when *K* = 4 and *K* = 5 population clusters were tested, the observed admixture patterns of the Miyi chicken and the Pengxian yellow chicken which is two distinct population living in lowland areas have a different genetic background to other indigenous chickens, respectively. This discrepancy may reflect the species ground dwelling, territorial characteristics etc.

We then used Japanese quail (*Coturnix japonica*) [12] as an outgroup to detect introgression between RJFs and 15 domestic chicken populations (Additional file 1: Table S4). Intensive crossbreeding has occurred in the past, and intercrossing between divergent breeds and occasionally even with wild jungle fowls was widely practiced [13]. This crossing implies a history of introgression between domestic breeds and wild populations. Our population structure analyses indicate a recent history of mutual introgression between Tibetan chickens and other breeds, as well as between wild RJFs and domestic populations. Introgression signatures were widespread in the domestic chicken populations. Archaeological discoveries in the Indus Valley and in Hebei Province, China, suggest that chickens were probably domesticated from the red jungle fowl as early as 5400 BC [14]. During the domestication of these local Chinese chicken breeds, chickens were often allowed to roam in a free-range state, and recurrent admixture between wild RJFs and domesticated chickens was very common, especially among breeds raised in mountainous areas; More introgression from RJFs to Tibetan chickens (the average number of IBDs is 3144) were found compared to others (the average number of IBDs is 2150). Thus, the most likely explanation for the dispersion pattern seen in domestic individuals is a long history of genetic exchange between RJFs and chickens. We found evidence of admixture between Tibetan chickens and other domesticated individuals possibly due to the influence of increased human activity with K changing progressively from 2 to 7. These crosses imply a history of introgression between domestic breeds and wild populations. Pairwise *D*-statistics based on ABBA and BABA SNP frequency differences and tracts of identity by descent (IBD) revealed that RJFs are closer to Xishuangbanna game fowls and Jinyang silky fowls than to other lowland breeds (Additional file 1: Table S5).

### Genome-wide selective sweep signals in domestic fowls

**Fig. 2:**
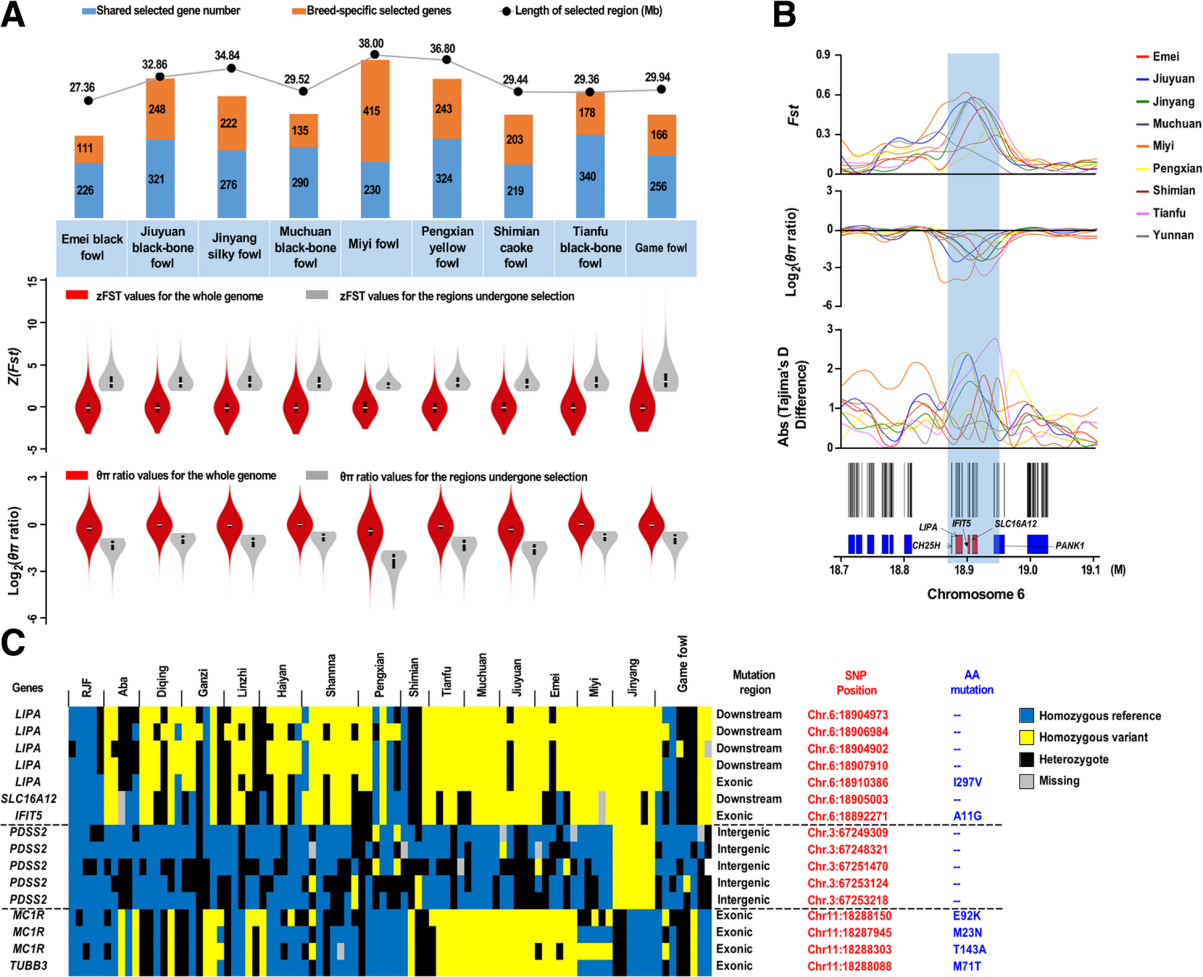
Genome-wide selection in domestic chicken breeds. **a** Genomic regions with strong selective sweep signals among domestic chicken breeds. The length of the genomic region with strong selective sweep signals is shown for each breed. The numbers of shared genes and breed-specific genes in the region are also listed. “Shared selected gene number” is the number of genes selected in ≥2 chicken breeds. Violin plots show the *zF*_*ST*_ values and the *θ*_*π*_ ratio for the regions of the breeds that have undergone selection (grey) versus the whole genome (red) (bottom panels). The vertical black boxes denote the interquartile range (IQR) between the first and third quartiles (25th and 75th percentiles, respectively), and the white point inside denotes the median. **b** Genomic region with strong selective sweep signals on chromosome 6 in the domestic chicken breeds. The values of *F*_*ST*_, log_2_(*θπ* ratio) and absolute Tajima’s *D* difference are plotted. The region with *zF*_*ST*_ ≥ 5 is shaded. The genome annotations are shown at the bottom (black bar: coding sequences; blue bar: genes) and the boundaries of *SLC16A12*, *LIPA, CH25H, PANK1* and *IFIT5* are marked in red. **c** A heatmap shows the selected SNP sites and the region containing *SLC16A12*, *LIPA, PDSS2, MC1R, TUBB3* and *IFIT5* from RJFs, Tibetan chickens and other domestic chicken breeds. Mutations with amino acid changes are shown on the left

To accurately detect the genomic footprints left by the intense artificial selection pressure, we performed pairwise comparisons of the genome-wide variation between the wild fowls and 10 geographically close but phenotypically diverse domestic breeds. After reviewing the distribution (Additional file 1: Figure S8), we concluded that 40 kb was the most appropriate window size because this size yielded few windows with ≤20 SNPs and retained a theoretically appropriate length to detect smaller sweeps. We identified genomic regions (with a total size of 27.36–38.00 Mb, corresponding to 2.61 to 3.63% of the genome and containing 337–645 genes per breed) with strong selective sweep signals in each breed that also exhibited significant differences (*p* < 10^− 16^, Mann-Whitney *U* test) in the log_2_(*θ*_*π*_ ratio) and *F*_ST_ value compared with the wild RJF genomic background (Fig. 2a; Additional file 1: Figures S9 and S10). Many genes in genomic regions with strong selective sweep signals are involved in growth, metabolism, immunity, behavior and reproduction (Additional file 1: Table S6) and may potentially contribute causally to phenotypic changes during selective breeding. We have identified a total of 74 genes that had strong selective sweep signals and were shared by more than five chicken breeds. Strikingly, five genes (*CH25H, PANK1*, *LIPA*, *SLC16A12* and *IFIT5*) in an 80-kb region (18.86–18.94 Mb) of chromosome 6 were under positive selection in at least five of the domestic populations, which had the highest values of *F*_*ST*_, at 0.54–0.61, and of z*F*_*ST*_, at 4.02–7.60 (*p* < 0.001) (Fig. 2b and c, Additional file 2).

LIPA catalyzes the intracellular hydrolysis of cholesteryl esters and triglycerides in hepatocytes and macrophages, and a *LIPA* deficiency is associated with abnormal lipid deposition in multiple organs in humans [15]. *IFIT5* confers antiviral defense by disrupting protein-protein interactions in the host translation initiation machinery [16], and its expression is induced in ducks upon infection with influenza virus [17]. The selective sweep of genes involved in lipid and glucose metabolism and immune defense [18, 19] may be responsible for the dramatic phenotypic changes that are of economic value in domestic chickens, such as meat yield and disease resistance. *SLC16A12* is a member of the carboxylic acid transporter family, essential for the establishment and/or maintenance of homeostasis in the lenses and kidneys. Mutations in *SLC16A12* lead to deficiency in the transportation of metabolites, contributing to the development of cataracts and renal glycosuria in humans [20]. Recent studies have demonstrated that cholesterol 25-hydroxylase (Ch25h) is an interferon-inducible protein that can inhibit the replication of many enveloped viruses [21]. Pantothenate kinase (PanK), encoded by the gene *PANK1*, is the rate-determining enzyme in coenzyme A (CoA) biosynthesis [22]. Collectively, *LIPA*, *SLC16A12* and *IFIT5* may constitute the main genetic contributors to meat yield and disease resistance in modern chickens. This potential candidate region on chromosome 6 containing *LIPA*, *SLC16A12* and *IFIT5* could be considered a main genetic contributor to chicken domestication.

### Phenotypic traits analysis

#### Silky feathers

We observed 204 unique selected genes in a 20.7-Mb region on the chromosomes of Jinyang silky fowls, with the highest *F*_*ST*_–window occurring at 10.02–10.38 Mb (*F*_*ST*_ = 0.69, *zFst* = 7.68, *p* < 0.001). The top ten selected genes included *CACNA2D1*, *GRM8*, *PDE7B*, *NECAB2*, *CAMK2D*, *SLC38A8*, *PDSS2*, *ZBTB43*, *NFATC3* and *ASCC3*. It should be noted that *PDSS2* (*F*_*ST*_ = 0.60, *zFst* = 6.44, *p* < 0.001) is a gene previously established as the causal gene for the silky-feather phenotype [23]. The causative mutation is located 103 base pairs upstream of the coding sequence of *PDSS2*. In addition, in this study, we found a SNP (chr3:67,304,974) located in the coding region of *PDSS2* that is specific for this breed, and the function of this mutation has not been reported yet. Further studies are needed to explore the function of this mutation.

#### Body weight

We detected the strongest selective sweep, reaching a z*F*_*ST*_ score of 9.67 (*F*_*ST*_ = 0.70, *p* < 0.001), in Muchuan black-boned fowl; the sweep was located in the upstream non-coding region of the *SMPD3* gene. *SMPD3* controls postnatal growth and development, and inactivation of *SMPD3* is associated with skeletal deformities [24]. The represented genes encoding myofibrillar proteins (*MYH1E*, *MYH1C* and *MYPN*) also had high z*F*_*ST*_ scores. The highest differentiation peak in the Shimian Caoke fowl occurred in a region containing the *GLI3* gene (*F*_*ST*_ = 0.66, z*F*_*ST*_ = 7.04, *p* < 0.001), which plays a role in the patterning of the brain and limbs [25]. These genes are associated with growth, consistent with the fact that these two chicken breeds have the greatest body weight at 180 days of age among all the included breeds (Additional file 1: Table S7).

#### Egg production

Among all 10 chicken breeds we examined, Tianfu black-boned fowls lay the greatest number of eggs (*n* = 102.46) at 300 days of age (Additional file 1: Table S7). We detected a selected region for laying traits that harbored the *KIF18A* gene and had the highest differentiation peak (*F*_*ST*_ = 0.57, z*F*_*ST*_ = 7.76, *p* < 0.001). This is not surprising, given a recent report that *KIF18A* is required for mitotic progression during germline development [26]. We also found that *KIF18A* was under selection in Pengxian yellow fowl (*Fst* = 0.514, *zFst* = 4.668) which ranked 2nd on egg numbers. Additionally, a highly differentiated peak occurred in a region containing *LIN28* (*F*_*ST*_ = 0.44, z*F*_*ST*_ = 5.87, *p* < 0.001), which encodes a microRNA-binding protein [27] that is also known as a stem cell factor [28]. Testis-expressed gene 11 (*Tex11*) (*F*_*ST*_ = 0.23, z*F*_*ST*_ = 2.59, *p* < 0.001), which affects testicular size in Australian Brahman cattle [29], also exhibited a selection signal in our analysis.

#### Black skin and bones

Three breeds in this study (the Muchuan black-boned fowl, the Tianfu black-boned fowl and the Jiuyuan black-boned fowl) have black skin and bones, whereas the other chicken populations have white skin and bones. The inferred genome variation that is subject to a selective sweep consists of a set of 22 genes that are mainly located on chromosome 11 (Additional file 1: Table S8). One of these candidate genes, melanocortin-1 receptor (*MC1R*), plays a crucial role in melanocyte development, proliferation and differentiation and is associated with coat color in many mammals, including coloration in Holstein dairy cattle and eumelanin production in rats [30]. In chicken, *BMP7* was found to be upregulated in hyperpigmentation of the visceral peritoneum (HVP) affected chickens [31]. By comparing black-boned chickens with non-black-boned domestic fowls, we found that the genes detected by Dorshorst et al., such as *EDN3* (*F*_*ST*_ = 0.153, z*F*_*ST*_ = 5.23, *p* < 0.001) and *TUBB1* (*F*_*ST*_ = 0.156, z*F*_*ST*_ = 5.33, *p* < 0.001), also showed high differentiation peaks. They found that a complex genomic rearrangement involving the endothelin 3 locus causes dermal hyperpigmentation in the Chicken [32]. In addition to these genes, our results suggested that *AEBP2*, *CHMP2B*, *PLEKHA5*, *PIT-1*, *MC1R* and *TUBB3* might also be associated with the trait of black bones, feathers and skin in birds (Fig. 2c).

#### Aggressiveness

**Fig. 3:**
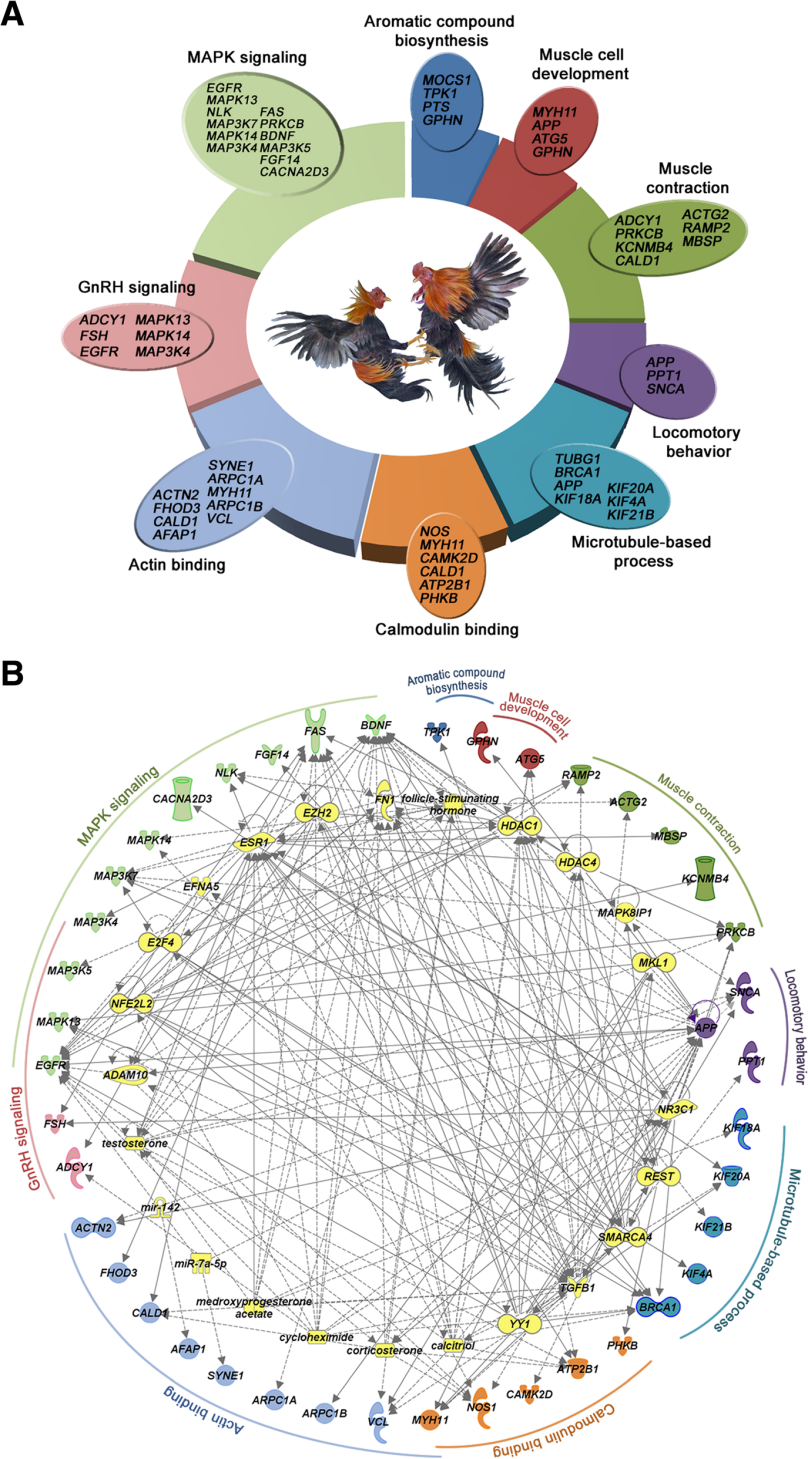
Aggressiveness-related genes and associated pathways. **a** Whole-genome comparison between eight game fowls and five RJFs reveals the enrichment of 46 selected genes involved in nine biological processes and pathways that contribute to the aggressiveness behavior. **b** Ingenuity Pathway Analysis (IPA) networks of aggressiveness-associated pathways. Aggressiveness proteins are represented on the outer circle with the corresponding pathways. Predicted regulators are represented on the inner circle. Different shapes denote the nature of molecules

The Xishuangbanna game fowl is a famous game breed originating in Yunnan, China. However, the genes underlying the trait of aggressiveness remain unknown. It seems that positive selection of game fowls leads to convergence of neural activity, and defects in relevant genes have been implicated in the pathogenesis of Alzheimer’s and Parkinson’s diseases. For example, *APP*, *SNCA* and *PPT1* are involved in neural activity, and defects in those genes have been implicated in the pathogenesis of Alzheimer’s disease, Parkinson’s disease, and infantile neuronal ceroid lipofuscinosis, respectively [33–35]. In addition, many genes related to muscle development and cardiovascular activity, such as *FGF14* [36], *VCL* [37], *MYH11* and *SYNE1* [38] were also found to be altered in the game breeds. The protein encoded by *MYH11* is a smooth muscle myosin belonging to the myosin heavy chain family. It functions as a major contractile protein, converting chemical energy into mechanical energy through the hydrolysis of ATP [39]. Effective production of mechanical energy would be advantageous to game fowls in combat. A network of the proteins encoded by specific genes (Fig. 3a, b) shows their involvement in pathways that are probably responsible for aggressiveness, including pathways related to aromatic compound biosynthesis, muscle cell development, muscle contraction, locomotor behavior, microtubule-based processes, calmodulin binding, actin binding, GnRH (Gonadotropin-releasing hormone) signaling and MAPK (mitogen-activated protein kinase) signaling.

### High-altitude adaptation in Tibetan chickens

To determine whether the Tibetan chicken has been subject to natural selective pressure for altitude adaptation, we explored the genomic landscape of population differentiation to identify candidate genes that potentially control high-altitude-specific adaptive traits present in Tibetan chickens. Our previous study found that there are at least three distinct clusters among the six geographically representative populations of Tibetan chickens: the chicken inhabiting Tibet and Qinghai (in cluster 3, TC1) were genetically closer to the RJF than to other domestic chickens, while the Tibetan chickens inhabiting Yunnan and Sichuan (clusters 1 and 2, TC2) were closer to the domestic populations and were split into two clusters [5].

We measured genome-wide variations and the frequency spectrum separately in Tibetan and lowland chickens (Additional file 1: Figure S11). A total of 158 and 117 candidate genes with selection signals were identified using sliding-window analysis in TC1 and TC2 respectively (Additional file 2). These genes were involved in 25 categories (Additional file 1: Table S9). We discovered five genes under selection in all Tibetan chickens (*TRIT1, HPCAL4*, *NT5C1A*, *LOC419677*, and *HEYL*) in a small region (40 kb) of chromosome 23 that showed high *zF*_*ST*_ and low log_2_(*θπ* ratio) values (Additional file 2); these genes could help explain the local adaptation of Tibetan fowls. *TRIT1* is a tumor suppressor, and its expression can decrease cell growth [40], which could be responsible for the dwarf phenotype of Tibetan chickens. In addition, the protein encoded by the *HPCAL4* gene is a Ca^2+^-binding protein highly similar to human hippocalcin protein and hippocalcin-like 1 protein. Human cytosolic 5′-nucleotidase is expressed at high levels in skeletal and heart muscle [41] and is involved in purine, pyrimidine, nicotinate and nicotinamide metabolism. Cytosolic 5′-nucleotidase 1A (*NT5C1A*) is also important in regulation of body fluid levels. This gene has been identified among a set of transcripts that are differentially expressed during high-altitude acclimatization in the Indian Air Force and Army Aviation Corps populations and is postulated to allow for more efficient oxygen utilization [42]. *HEYL* is a member of the Hairy-related transcription factor (HRT) family. HRT proteins may regulate specific sets of cardiac genes by modulating the function of GATA proteins and other cardiac transcriptional activators in a signal-dependent manner [43]. Tibetan chickens inhabiting high-altitude locales have increased red blood cell counts, blood oxygen affinity and hemoglobin concentrations, as well as decreased mean corpuscular volume, to overcome the harsh extremes of their environment [44]. Therefore, *NT5C1A* and *HEYL* may be involved in the high-altitude adaption of oxygen delivery in Tibetan chickens. Several candidate genes in the calcium-signaling pathway may be involved in the hypoxia adaptation experienced by Tibetan chickens [45]. Additionally, few of the positively selected genes were involved in the hypoxia-inducible factor 1 signaling pathway, which is primarily utilized in response to hypoxia in mammals such as humans [46, 47], Tibetan Mastiffs [48, 49] and horses [50]. In this study, these genes were mainly associated with energy metabolism, immune response, growth regulation and skeletal muscle development. These biological processes in particular are involved in GTPase regulation activities, suggesting the importance of energy metabolism for maintenance of proper body temperature in Tibetan chicken populations.

We next searched for mutations in coding sequences that have become fixed or nearly fixed in Tibetan chickens. We first searched for nonsense mutations as obvious candidates of functional significance that may have contributed to rapid evolution in Tibetan chickens. However, none of the candidates occurred at a high frequency in these Tibetan chicken lineages. Then, we screened the individual sequence data for the presence of mutations that showed a marked allele frequency difference between Tibetan chickens and other domestic chickens (greater than 80% in one group, less than 20% in the other). Differences in allele frequencies between the two groups were analyzed using Pearson’s chi-squared test. The *p* values from Pearson’s chi-squared test were < 0.001 for all alleles. Eleven SNPs located in coding regions were found to have extreme differences in allele frequencies between Tibetan chickens and other domestic chickens, and only three genes, namely, *PKD2L1, EVI5* and *ZDHHC9*, consisted of nonsynonymous mutations (Additional file 1: Figure S12A).

*PKD2L1* encodes a member of the polycystin protein family that is an integral membrane protein involved in cell-cell/matrix interactions. Animals lacking the *PKD2L1* gene are completely devoid of taste responses to sour stimuli [51]. On the Tibetan plateau, wet hulless-barley distillers’ grains (WHDG) that contain a large amount of acid (such as acetic acid, propionic acid, butyric acid and lactic acid) are produced annually and commonly used in combination with drier feedstuffs to feed Tibetan domesticated animals [52]. WHDG feed is potentially part of why *PKD2L1* has been under selection in Tibetan chickens. *EVI5* is involved in the regulation of GTPase activity. Through genetic adaptations, Tibetan chickens may have developed effective strategies to cope with the acidity of their feed or to cope with the alpine environment in high-altitude regions. Mutations in the coding region of *EVI5* aligned with the orthologous protein sequences from 4 other vertebrates were shown in Additional file 1: Figure S12B. *ZDHHC9* encodes a palmitoyltransferase of NRAS and HRAS, and its mutation is associated with X-linked mental retardation [53]. Further studies are needed to confirm whether *ZDHHC9* is involved in high-altitude adaption.

In summary, we found that genes associated with high-altitude adaptation in Tibetan chickens were mainly involved in energy metabolism, body size maintenance and digestion, which could be related the chilly climate of the highlands, the high body temperature characteristic of birds, the dwarf phenotype of Tibetan chickens, and adaption to available food sources. Additionally, we believe that birds can adapt to hypoxic stimuli by changing the expression levels of genes. Transcriptome data from lowland and Tibetan chickens are needed to further explore the high-altitude hypoxia adaption of birds.

## Conclusion

By analyzing multiple individual genomes representing various chicken populations and breeds, this study reveals a large and complex landscape of genetic diversity and selective sweeps that occurred during the domestication of chickens. Comparison of the genome sequences of red jungle fowls, nine lowland domestic chicken breeds and Tibetan chickens provided insights into the distinct evolutionary scenarios occurring under artificial selection for agricultural production and under natural selection for success at a high altitude. Several genes were identified as candidates for chicken economic traits and other phenotypic traits. Our results indicate that Tibetan chickens evolved in adaptation to a high-altitude alpine environment during their long history of living at high altitudes.

## Methods

### Genome sequencing, analysis of the population structure and evolutionary history

The whole genomes of 86 domestic chickens including 50 lowland chickens from 9 phenotypically diverse breeds ([Emei black fowl, Emei, 400 m altitude], [Jiuyuan black fowl, Jiuyuan, 900 m altitude], [Jinyang silky fowl, Jinyang, 460 m altitude], [Muchuan black-boned fowl, Muchuan, 500 m altitude], [Miyi fowl, Miyi, 1400 m altitude], [Pengxian yellow fowl, Pengxian, 800 m altitude], [Tianfu black-boned fowl, Chengdu, 540 m altitude], [Shimian Caoke fowl, Shimian, 790 m altitude] and Xishuangbanna game fowl) in China and 36 Tibetan chickens from 6 Qinghai-Tibetan Plateau localities ([Aba, Sichuan Province, 3300 m altitude], [Ganzi, Sichuan Province, 3390 m altitude], [Diqing, Yunnan Province, 3280 m altitude], [Haiyan, Qinghai Province, 3260 m altitude], [Linzhi, Tibet, 3100 m altitude] and [Shannan, Tibet, 3700 m altitude]) (Additional file 1: Table S1)), together with 5 red jungle fowls (RJFs), were used for comparative population genomics. Among them, the genomes of Xishuangbanna game fowl and RJFs were downloaded from NCBI (GenBank accession number PRJNA241474). Description and quantification of the phenotypic diversity of each breed are provided in Additional file 1: Table S7. The whole genomes of chickens were sequenced on the Illumina HiSeq 2000 platform. We generated sequencing libraries using a TruSeq Nano DNA HT Sample Preparation Kit (Illumina, San Diego, CA, USA) following the manufacturer’s instructions, and index codes were added to attribute sequences to each sample. Briefly, the DNA sample was fragmented to a size of 350 bp by sonication, and the DNA fragments were then end polished, A-tailed, and ligated with the full-length adapter for Illumina sequencing with further polymerase chain reaction (PCR) amplification. The sequencing data have been submitted to NCBI Sequence Read Archive (SRA) with the accession number SRP067615. Approximately 96.30% of high-quality paired-end reads were mapped to the chicken reference genome with an average coverage depth of 18.06-fold for each individual using BWA software [54]. We first examined individual SNPs independently confirmed by both SAMtools [14] and GATK [55]. The methods for genome sequencing, sequence filtering, data analysis, SNP calling, and insertion and deletion (indel) calling can be found in our previous study [5]. To evaluate LD decay, we used Haploview [56] to calculate the coefficient of determination (*r*^2^) between each pair of loci. The average *r*^2^ was calculated for the pairwise markers in a 40-kb window and averaged across the whole genome. To determine the most appropriate window size, capable of yielding few windows with ≤20 SNPs while retaining a theoretically appropriate length to detect smaller sweeps, we computed the number of bins with ≥20 SNPs in 1-Mb, 500-kb, 200-kb, 100-kb, 40-kb, 20-kb and 10-kb windows (Additional file 1: Table S10). The window size with the highest proportion of bins with ≥20 SNPs was considered the best one to use. Population structure analysis with a full maximum-likelihood approach was performed using *frappe* [11].

### Segmenting the chicken genome into isochores

The chicken genome (Gallus_gallus 4) was first split into single chromosomes. isoSegmenter (Version 1.5.1) was used to identify isochores on each chromosome, with all parameters on default settings except window size (https://github.com/bunop/isoSegmenter). More specifically, we calculated the standard deviation (SD) of GC for fixed window sizes (w) ranging from 10 kb to 750 kb for all isochores comprising at least four windows. The smaller window size was selected when a plateau began to emerge across increasing window sizes [57].

### Introgression analysis – *D*-statistics (ABBA-BABA tests) and identity by descent (IBD)

To detect introgression between RJFs and the 10 domestic chicken breeds, we computed *D*-statistics based on ABBA and BABA SNP frequency differences using the following expression [58]:

$$ D\left({P}_1,{P}_2,{P}_3,\mathrm{Japanese}\ \mathrm{quail}\right)=\frac{\sum_{i=1}^n\left(1\hbox{-} {\widehat{p}}_{i1}\right){\widehat{p}}_{i2}{\widehat{p}}_{i3}-{\widehat{p}}_{i1}\left(1-{\widehat{p}}_{i2}\right){\widehat{p}}_{i3}}{\sum_{i=1}^n\left(1-{\widehat{p}}_{i1}\right){\widehat{p}}_{i2}{\widehat{p}}_{i3}+{\widehat{p}}_{i1}\left(1-{\widehat{p}}_{i2}\right){\widehat{p}}_{i3}} $$

where P1, P2, P3 and Japanese quail (P4) are the four different populations under comparison: P1 (one domestic chicken population) and P2 (another domestic chicken population), are sister taxa, P3 comprises RJFs and P4 (Japanese quail) is outgroup. The sequence-based genotypes were phased from the 91 samples, and tracts of identity by descent (IBD) between the two chicken populations were inferred with BEAGLE 4.1 [59].

### Sweep analysis

The selective sweep screen was performed using a method that includes the sequence diversity statistic (*θ*_π_) [60], the population differentiation statistic (*F*_ST_) [61] and Tajima’s *D* value [62] using a 40-kb window with a 20-kb step. To detect characteristics that have undergone selection associated with high altitude or domestication, we measured the genome-wide variation between the highland (Qinghai-Tibetan Plateau) and lowland groups (Sichuan Basin) and the genome-wide variation between the domesticated breeds and the RJFs. Because sex chromosomes and autosomes are subjected to different selective pressures and have different effective population sizes, the Z-transformed population differentiation (z*F*_*ST*_) [63] was estimated only for the autosomes. The formula used to calculate z*F*_*ST*_ was as follows: z(*F*_*ST*_) = (*F*_*ST*_ - μ(*F*_*ST*_))/σ(*F*_*ST*_), where *μ* is the mean of *F*_*ST*_ and σ is the standard deviation of *F*_*ST*_*.* The genome signature with significantly high *F*_*ST*_ (corresponding to the top 5% level) and *θ*_π_ ratio (*θ*_π, lowland population_/*θ*_π, Tibetan population_, top 5% level presented) values were identified as extensively diversified. The gene annotation is performed in the selected genomic regions according to the reference chicken genome. Genes were submitted to DAVID (https://david.ncifcrf.gov/) for enrichment analysis of the significant overrepresentation of GO biological process (GO-BP) and molecular function (GO-MF) terms, as well as InterPro domains and KEGG pathways. In all tests, the whole set of known chicken genes was defined as the background, and *p* values (i.e., EASE scores), indicating the significance of the overlap between various gene sets, were calculated using a modified Fisher’s exact test corrected by the Benjamini-Hochberg procedure. Only GO-BP, GO-MF, KEGG pathway or InterPro domain terms with a *p* value less than 0.05 were considered significant and included on the list. For aggressiveness of game fowls, we first detected genes selected for game fowls, and then these candidate genes were subjected to Ingenuity Pathway Analysis (IPA) network analysis [64].

## Additional files

Additional file 1: This file includes Figures S1 to S12 and Tables S1 to S10. (DOCX 17545 kb)

Additional file 2: Lists of information containing all the positively selected genes in chicken breeds. (XLSX 1018 kb)

## Abbreviations

ATP: Adenosine triphosphate
bp: Base pairs
DNA: Deoxyribonucleic acid
GO: Gene ontology
IBD: Identity by descent
indels: Insertion/Deletions
IPA: Ingenuity Pathway Analysis
LD: Linkage disequilibrium
m: Meter
NCBI: National Center for Biotechnology Information
PCR: Polymerase chain reaction
RJF: Red jungle fowl
SNP: Single nucleotide polymorphism
