# Supplementary material for: Population genomics identifies patterns of genetic diversity and selection in chicken
Source: BMC Genomics. 2019 Apr 2;20:263. doi: 10.1186/s12864-019-5622-4 (PMC6446315; doi:10.1186/s12864-019-5622-4)
Supplement: Supplementary file 1 — This file includes Figures S1 to S12 and Tables S1 to S10. (DOCX 17545 kb) [file 12864_2019_5622_MOESM1_ESM.docx]

**Table S1** Samples used for whole-genome analysis.

|  | Type | Breed | Population | Altitude | Number of chickens | Total SNP (Millions) |
| --- | --- | --- | --- | --- | --- | --- |
| Domestic fowls | Lowland chicken | Emei black fowl | Emei | 400m | 6 | 6.6 |
|  |  | Jiuyuan black-bone fowl | Jiuyuan | 900m | 5 | 6.51 |
|  |  | Jinyang silky fowl | Jinyang | 460m | 6 | 6.63 |
|  |  | Muchuan black-bone fowl | Muchuan | 500m | 5 | 6.69 |
|  |  | Miyi fowl | Miyi | 1400m | 5 | 5.27 |
|  |  | Pengxian yellow fowl | Pengxian | 800m | 6 | 6.25 |
|  |  | Shimian caoke fowl | Shimian | 790m | 4 | 5.63 |
|  |  | Tianfu black-bone fowl | Tianfu | 540m | 5 | 6.74 |
|  |  | Xishuangbanna Game fowl^1^ | Game fowl | 1500m | 8 | 7.11 |
|  | Highland chicken | Tibetan fowl | Aba | 3300m | 5 | 6.69 |
|  |  | Tibetan fowl | Diqing | 3280m | 6 | 7.28 |
|  |  | Tibetan fowl | Ganzi | 3390m | 6 | 6.93 |
|  |  | Tibetan fowl | Linzhi | 3100m | 5 | 6.74 |
|  |  | Tibetan fowl | Haiyan | 3260m | 6 | 7.05 |
|  |  | Tibetan fowl | Shannan | 3700m | 8 | 7.52 |
| Wild fowls | Red jungle fowl | Red jungle fowl^2^ | RJF |  | 5 | 6.72 |

^1, 2^ **indicate that the sequences were downloaded from NCBI.**


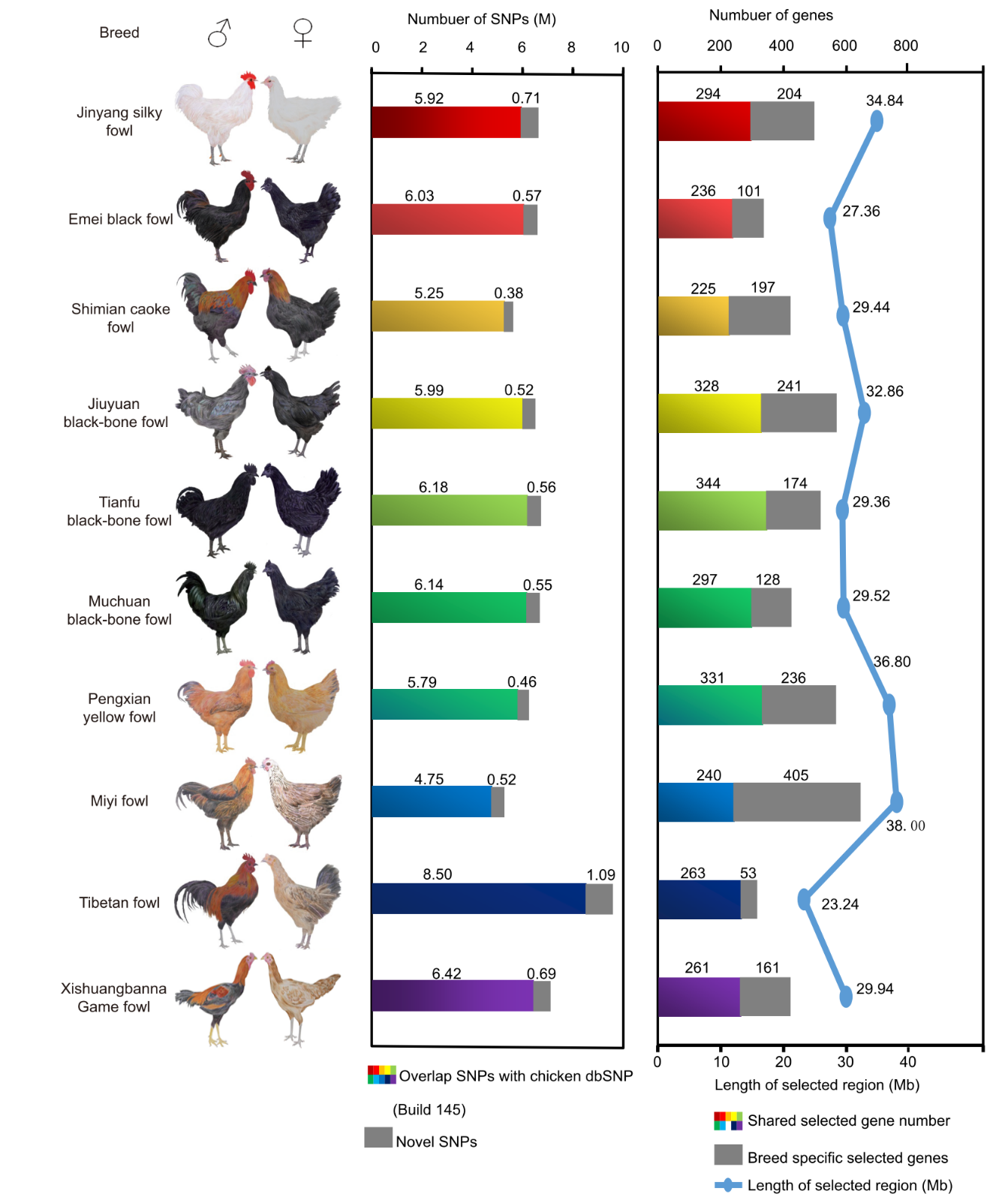


**Figure S1** Numbers of SNPs identified in ten domestic chicken breeds.

**Table S2** The frequency distribution of domestic chicken breed/population specific SNPs.

| Breed/  Population | Cumulative frequency | | | | | | | | | |
| --- | --- | --- | --- | --- | --- | --- | --- | --- | --- | --- |
|  | 0.1 | 0.2 | 0.3 | 0.4 | 0.5 | 0.6 | 0.7 | 0.8 | 0.9 | 1 |
| Pengxian | 377 | 662 | 874 | 1,031 | 1,202 | 1,261 | 1,305 | 1,344 | 1,367 | 1,398 |
| Jinyang | 523 | 903 | 1,167 | 1,304 | 1,496 | 1,561 | 1,629 | 1,677 | 1,709 | 1,727 |
| Emei | 990 | 1,579 | 1,892 | 2,105 | 2,310 | 2,361 | 2,419 | 2,460 | 2,489 | 2,511 |
| Jiuyuan | 643 | 1,017 | 1,214 | 1,366 | 1,480 | 1,533 | 1,589 | 1,618 | 1,649 | 1,665 |
| Muchuan | 631 | 1,002 | 1,225 | 1,366 | 1,467 | 1,510 | 1,566 | 1,609 | 1,639 | 1,654 |
| Miyi | 803 | 1,233 | 1,460 | 1,674 | 1,838 | 1,894 | 1,972 | 2,030 | 2,066 | 2,123 |
| Shimian | 757 | 1,361 | 1,429 | 1,570 | 1,740 | 1,779 | 1,843 | 1,861 | 1,875 | 1,908 |
| Tianfu | 643 | 1,035 | 1,262 | 1,394 | 1,514 | 1,564 | 1,603 | 1,633 | 1,644 | 1,651 |
| Aba | 984 | 1,529 | 1,851 | 2,045 | 2,159 | 2,225 | 2,296 | 2,334 | 2,364 | 2,379 |
| Diqing | 837 | 1,361 | 1,709 | 1,889 | 2,092 | 2,160 | 2,208 | 2,235 | 2,254 | 2,270 |
| Ganzi | 903 | 1,361 | 1,615 | 1,798 | 1,993 | 2,055 | 2,092 | 2,122 | 2,143 | 2,169 |
| Linzhi | 993 | 1,694 | 1,995 | 2,175 | 2,308 | 2,362 | 2,417 | 2,456 | 2,481 | 2,499 |
| Qinghai | 895 | 1,447 | 1,804 | 2,047 | 2,300 | 2,376 | 2,434 | 2,494 | 2,529 | 2,561 |
| Shannan | 1,092 | 1,518 | 1,998 | 2,162 | 2,395 | 2,478 | 2,576 | 2,645 | 2,667 | 2,687 |
| Game fowl | 1,392 | 2,886 | 3,462 | 3,940 | 4,246 | 4,395 | 4,563 | 4,639 | 4,687 | 4,716 |

Pengxian, Pengxian yellow fowl; Jinyang, Jinyang silky fowl; Emei, Emei black fowl; Jiuyuan, Jiuyuan black fowl; Muchuan, Muchuan black-boned fowl; Miyi, Miyi fowl; Shimian, Shimian Caoke fowl; Tianfu, Tianfu black-boned fowl; game fowl, Xishuangbanna game fowl; Tibetan populations include Aba, Diqing, Ganzi, Linzhi, Haiyan and Shannan.

**
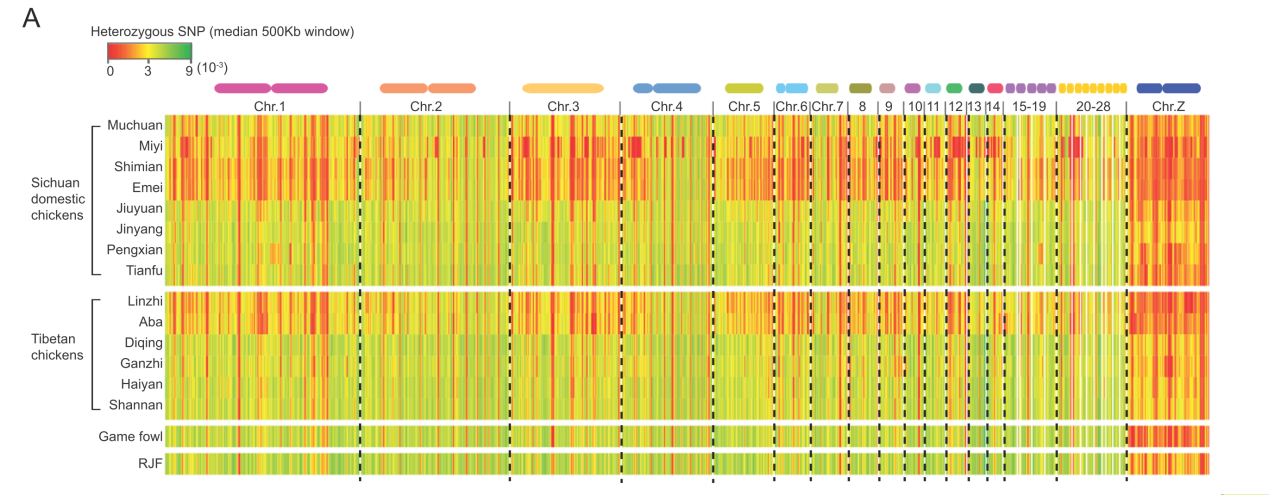
**


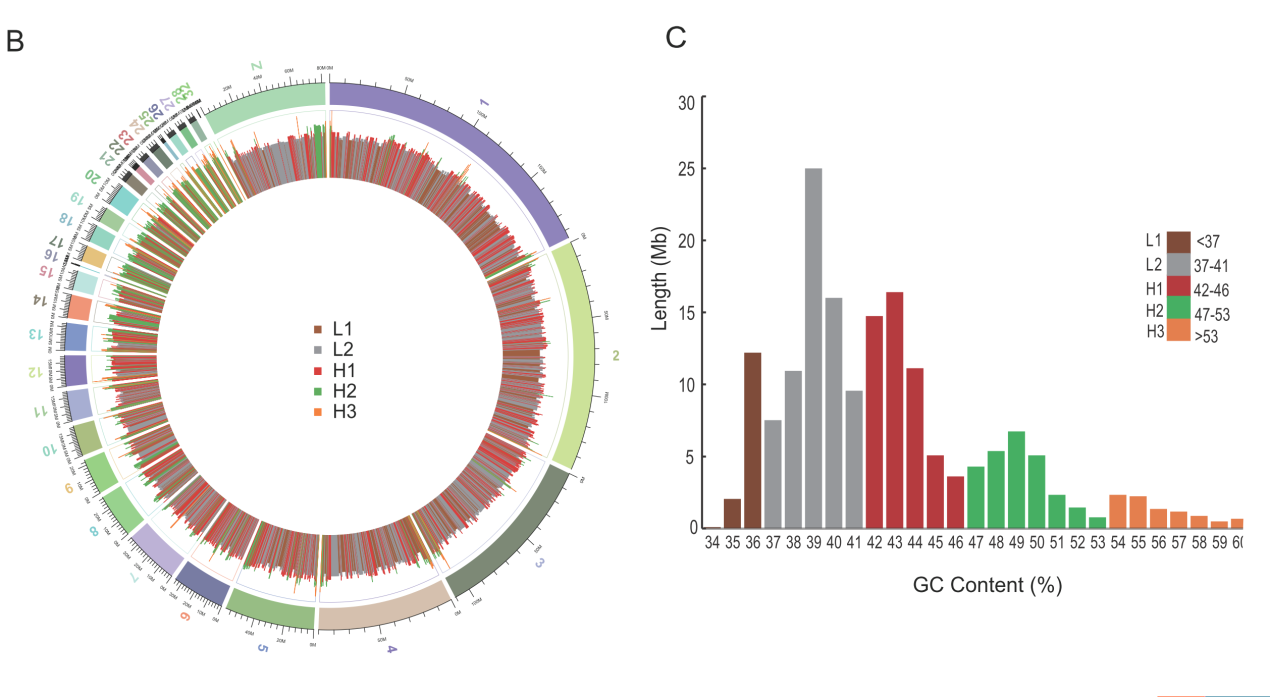


**Figure S2** (A) Heat map of the heterozygous SNP rate within each non-overlapping 500-kb window across the genomes. (B) Isochore profiles of all chicken chromosomes. (C) Distribution of isochores according to GC levels.


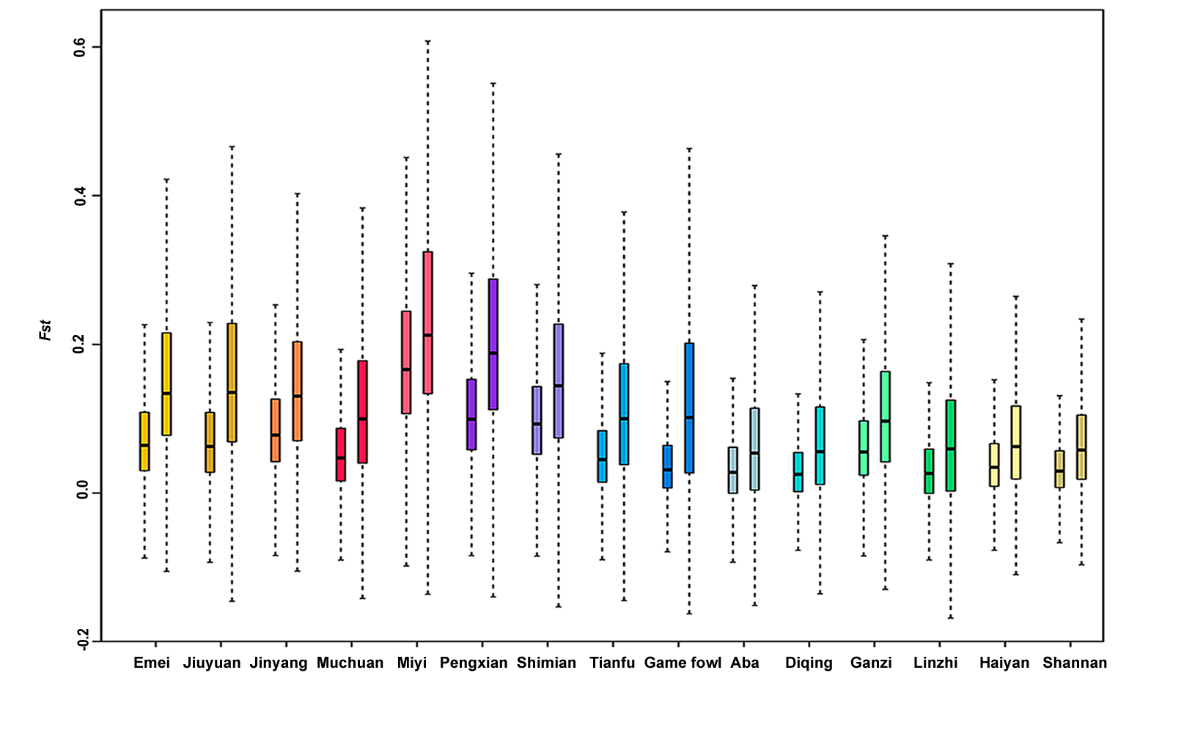


**Figure S3** Boxplot showing population genetic differentiation of autosomes (left) and Z chromosome (right) between each domestic chicken population and RJFs.


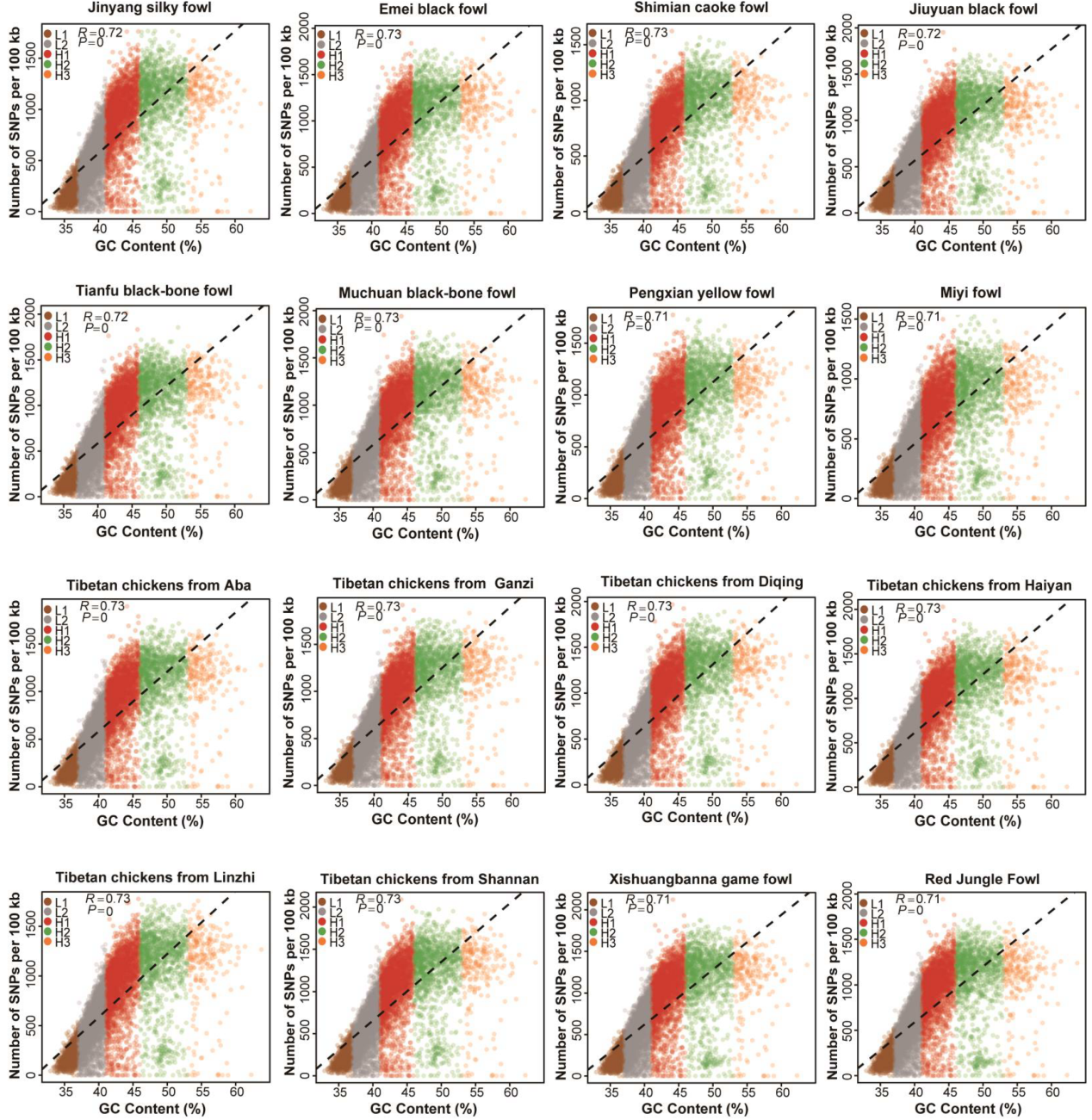


**Figure S4** Correlation between number of SNPs in 91 chickens and GC level in the isochores of the chicken genome.


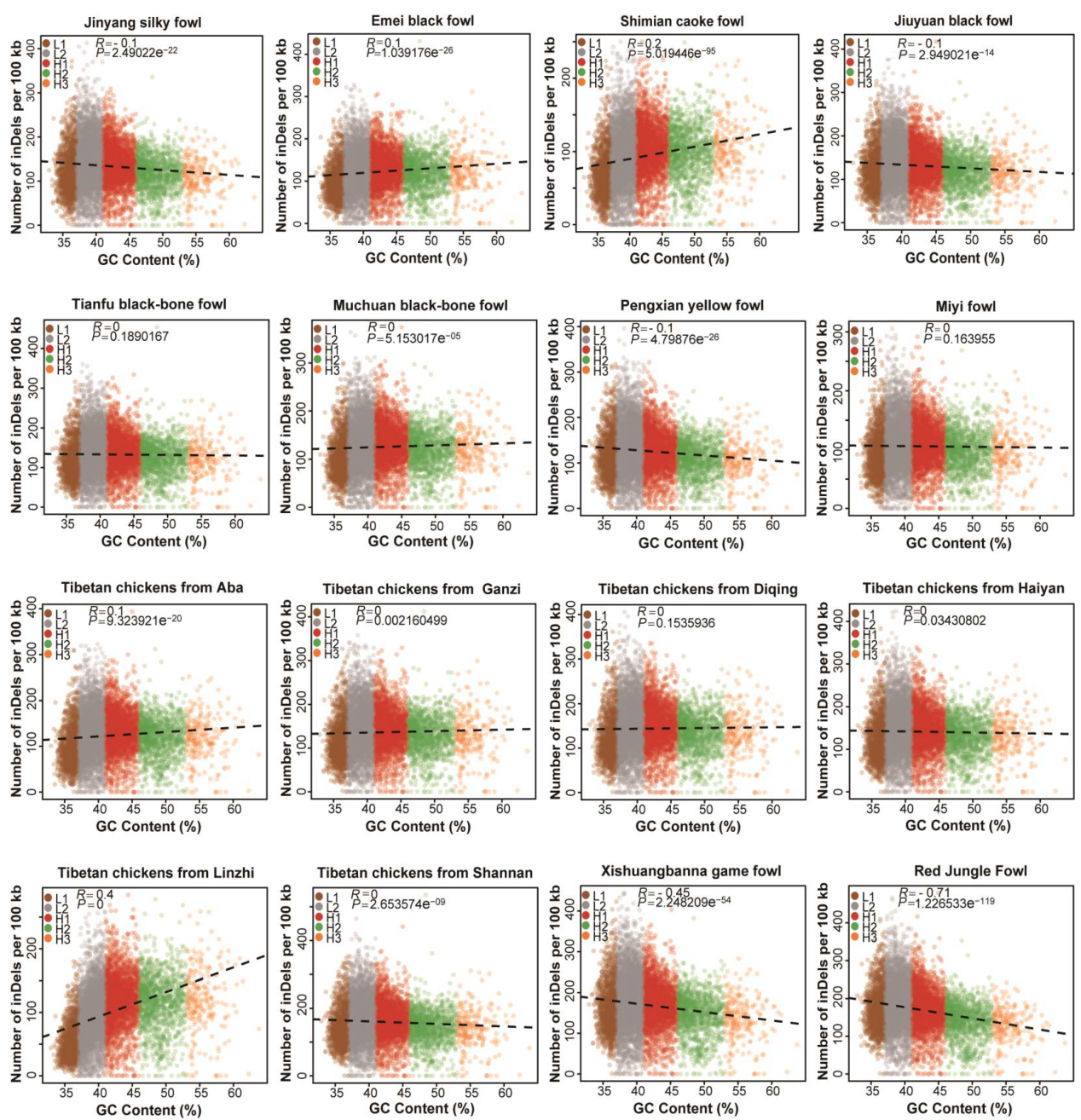
 **Figure S5** Correlation between number of indels (D) in 91 chickens and GC level in the isochores of the chicken genome.


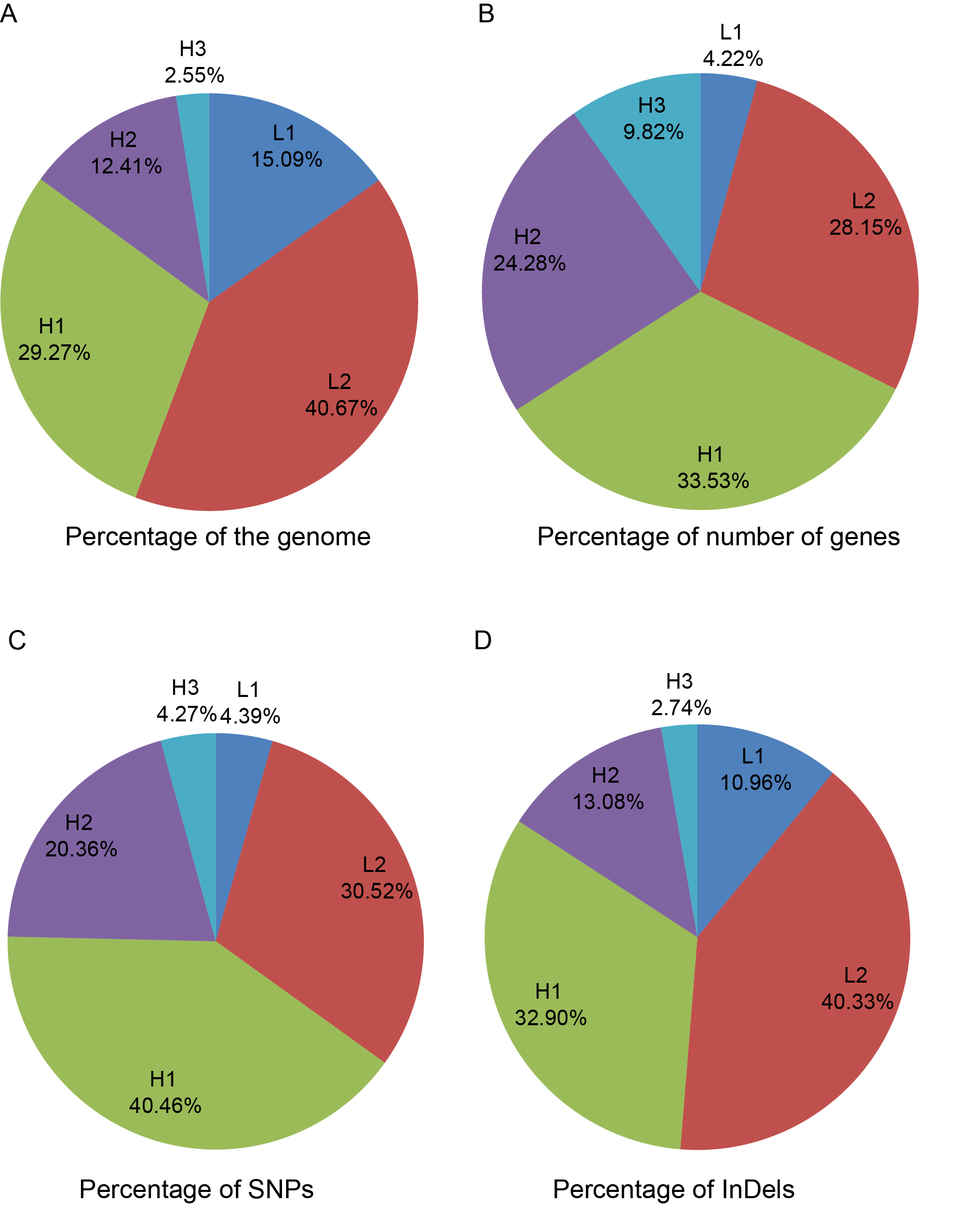


**Figure S6** (A) Isochore families of the chicken genome; (B) percentages of total number of genes, (C) SNPs and (D) indels in each isochore.

**Table S3** Distribution of SNPs and indels across isochores in 91 chicken genomes.

|  | L1 (15.09%) | | L2 (40.67%) | | H1 (29.27%) | | H2 (12.41%) | | H3 (2.55%) | |
| --- | --- | --- | --- | --- | --- | --- | --- | --- | --- | --- |
| Sample | SNP (%) | InDel (%) | SNP (%) | InDel (%) | SNP (%) | InDel (%) | SNP (%) | InDel (%) | SNP (%) | InDel (%) |
| LCEM1 | 4.21 | 6.15 | 29.25 | 35.02 | 40.17 | 37.17 | 21.59 | 17.56 | 4.78 | 4.09 |
| LCEM2 | 4.44 | 12.30 | 31.01 | 42.28 | 40.33 | 31.38 | 20.01 | 11.63 | 4.21 | 2.42 |
| LCEM3 | 3.83 | 5.28 | 27.84 | 31.12 | 40.77 | 38.96 | 22.64 | 19.99 | 4.91 | 4.64 |
| LCEM4 | 4.40 | 13.83 | 30.88 | 43.29 | 40.54 | 29.91 | 20.04 | 10.78 | 4.14 | 2.20 |
| LCEM5 | 3.37 | 5.00 | 25.24 | 29.42 | 41.38 | 39.36 | 24.56 | 21.28 | 5.45 | 4.93 |
| LCEM6 | 4.25 | 5.55 | 29.64 | 33.61 | 40.42 | 38.46 | 21.12 | 18.19 | 4.57 | 4.19 |
| LCJY1 | 4.52 | 8.12 | 30.59 | 39.39 | 40.21 | 35.33 | 20.29 | 14.09 | 4.39 | 3.07 |
| LCJY3 | 4.41 | 13.10 | 30.72 | 42.57 | 40.38 | 30.56 | 20.28 | 11.39 | 4.21 | 2.38 |
| LCJY5 | 4.43 | 13.93 | 31.03 | 43.30 | 40.44 | 29.82 | 19.98 | 10.77 | 4.13 | 2.18 |
| LCJY7 | 4.53 | 12.87 | 31.25 | 43.35 | 40.58 | 30.92 | 19.67 | 10.83 | 3.97 | 2.03 |
| LCJY8 | 4.54 | 13.08 | 31.15 | 43.25 | 40.52 | 30.71 | 19.76 | 10.88 | 4.03 | 2.08 |
| LCLS1 | 4.51 | 13.31 | 31.18 | 43.48 | 40.56 | 30.60 | 19.79 | 10.64 | 3.96 | 1.98 |
| LCLS2 | 4.48 | 12.87 | 31.26 | 43.49 | 40.52 | 30.76 | 19.73 | 10.84 | 4.01 | 2.03 |
| LCLS3 | 4.44 | 12.38 | 30.97 | 42.91 | 40.55 | 31.35 | 19.98 | 11.22 | 4.05 | 2.14 |
| LCLS4 | 4.48 | 13.55 | 31.36 | 43.67 | 40.43 | 30.22 | 19.73 | 10.55 | 4.00 | 2.01 |
| LCLS5 | 4.46 | 13.06 | 31.30 | 43.47 | 40.47 | 30.71 | 19.80 | 10.77 | 3.98 | 1.99 |
| LCLS6 | 4.47 | 12.91 | 31.43 | 43.62 | 40.49 | 30.80 | 19.63 | 10.65 | 3.99 | 2.02 |
| LCMC1 | 4.37 | 5.92 | 29.72 | 34.36 | 40.28 | 38.09 | 21.04 | 17.61 | 4.59 | 4.02 |
| LCMC2 | 4.38 | 5.89 | 30.51 | 34.92 | 40.29 | 38.10 | 20.48 | 17.26 | 4.34 | 3.83 |
| LCMC4 | 4.55 | 13.64 | 31.07 | 43.11 | 40.38 | 30.19 | 19.86 | 10.88 | 4.14 | 2.17 |
| LCMC5 | 4.42 | 8.08 | 30.48 | 38.96 | 40.35 | 35.30 | 20.43 | 14.48 | 4.32 | 3.17 |
| LCMC8 | 4.45 | 13.70 | 31.23 | 43.42 | 40.27 | 30.00 | 19.91 | 10.74 | 4.15 | 2.14 |
| LCMY1 | 4.35 | 6.12 | 29.99 | 35.69 | 40.44 | 37.82 | 20.71 | 16.65 | 4.51 | 3.72 |
| LCMY2 | 4.52 | 13.42 | 31.07 | 43.17 | 40.40 | 30.25 | 19.87 | 10.91 | 4.14 | 2.25 |
| LCMY3 | 4.27 | 11.07 | 30.00 | 40.20 | 40.37 | 32.35 | 20.85 | 13.44 | 4.51 | 2.94 |
| LCMY4 | 4.45 | 13.39 | 31.17 | 43.29 | 40.51 | 30.37 | 19.75 | 10.78 | 4.13 | 2.18 |
| LCMY6 | 4.20 | 5.26 | 29.56 | 33.07 | 40.64 | 39.07 | 21.09 | 18.49 | 4.51 | 4.11 |
| LCPX1 | 4.57 | 13.47 | 31.03 | 42.85 | 40.49 | 30.57 | 19.87 | 10.89 | 4.04 | 2.21 |
| LCPX2 | 4.51 | 13.18 | 31.24 | 43.22 | 40.48 | 30.52 | 19.61 | 10.84 | 4.15 | 2.24 |
| LCPX3 | 4.58 | 13.54 | 30.98 | 43.15 | 40.38 | 30.35 | 19.89 | 10.79 | 4.17 | 2.18 |
| LCPX4 | 4.63 | 13.48 | 31.01 | 43.22 | 40.53 | 30.51 | 19.83 | 10.77 | 4.00 | 2.02 |
| LCPX5 | 4.57 | 13.82 | 31.13 | 43.43 | 40.43 | 30.23 | 19.87 | 10.56 | 4.00 | 1.96 |
| LCPX6 | 4.57 | 13.85 | 31.44 | 43.63 | 40.26 | 30.09 | 19.79 | 10.48 | 3.95 | 1.95 |
| LCSM4 | 4.09 | 7.21 | 28.64 | 34.04 | 40.26 | 36.54 | 22.17 | 18.02 | 4.84 | 4.19 |
| LCSM5 | 3.81 | 4.89 | 27.37 | 30.01 | 41.14 | 39.79 | 22.78 | 20.64 | 4.90 | 4.67 |
| LCSM6 | 4.28 | 4.90 | 29.78 | 31.94 | 40.33 | 39.16 | 21.09 | 19.53 | 4.53 | 4.46 |
| LCSM8 | 4.48 | 12.93 | 30.97 | 43.01 | 40.32 | 30.74 | 20.07 | 11.08 | 4.16 | 2.24 |
| LCTF2 | 4.53 | 7.81 | 30.99 | 39.11 | 40.34 | 35.71 | 19.94 | 14.35 | 4.19 | 3.01 |
| LCTF3 | 4.44 | 14.47 | 31.05 | 43.34 | 40.49 | 29.42 | 19.88 | 10.62 | 4.15 | 2.15 |
| LCTF4 | 4.35 | 5.38 | 29.16 | 32.37 | 40.43 | 38.78 | 21.41 | 19.07 | 4.64 | 4.40 |
| LCTF6 | 4.51 | 12.11 | 30.75 | 41.56 | 40.49 | 31.62 | 20.07 | 12.14 | 4.17 | 2.56 |
| LCTF8 | 4.45 | 13.10 | 31.17 | 43.30 | 40.26 | 30.39 | 19.91 | 10.96 | 4.21 | 2.25 |
| RJF1 | 4.48 | 14.58 | 30.82 | 43.47 | 40.50 | 29.91 | 20.09 | 10.23 | 4.10 | 1.82 |
| RJF2 | 4.47 | 14.98 | 30.94 | 43.38 | 40.60 | 29.42 | 19.92 | 10.29 | 4.08 | 1.93 |
| RJF3 | 4.28 | 13.39 | 31.01 | 43.79 | 40.98 | 30.82 | 19.76 | 10.21 | 3.98 | 1.78 |
| RJF4 | 4.44 | 14.88 | 31.42 | 44.18 | 40.71 | 29.44 | 19.51 | 9.81 | 3.92 | 1.69 |
| RJF5 | 4.34 | 14.93 | 31.30 | 43.89 | 40.61 | 29.31 | 19.72 | 10.04 | 4.03 | 1.83 |
| TCAB1 | 4.28 | 7.23 | 29.84 | 37.93 | 40.48 | 36.19 | 20.93 | 15.28 | 4.48 | 3.36 |
| TCAB3 | 4.51 | 11.61 | 31.05 | 42.56 | 40.32 | 31.65 | 19.98 | 11.75 | 4.15 | 2.42 |
| TCAB5 | 4.27 | 6.40 | 30.24 | 36.52 | 40.40 | 37.49 | 20.63 | 16.05 | 4.46 | 3.54 |
| TCAB6 | 4.35 | 6.70 | 30.44 | 37.36 | 40.87 | 37.30 | 20.17 | 15.40 | 4.16 | 3.24 |
| TCAB7 | 4.51 | 13.63 | 31.09 | 43.20 | 40.22 | 30.10 | 19.98 | 10.87 | 4.20 | 2.20 |
| TCDQ1 | 4.46 | 13.41 | 31.03 | 43.31 | 40.45 | 30.35 | 19.90 | 10.75 | 4.17 | 2.18 |
| TCDQ2 | 4.16 | 4.83 | 29.41 | 31.99 | 40.42 | 39.56 | 21.36 | 19.22 | 4.66 | 4.41 |
| TCDQ3 | 4.49 | 12.69 | 31.02 | 42.97 | 40.40 | 30.90 | 19.94 | 11.17 | 4.15 | 2.27 |
| TCDQ4 | 4.48 | 12.73 | 30.93 | 42.32 | 40.37 | 30.90 | 20.02 | 11.61 | 4.20 | 2.43 |
| TCDQ5 | 4.30 | 9.87 | 30.01 | 40.05 | 40.15 | 33.28 | 21.00 | 13.74 | 4.55 | 3.05 |
| TCDQ6 | 4.43 | 7.12 | 30.77 | 38.09 | 40.18 | 36.44 | 20.32 | 15.15 | 4.29 | 3.21 |
| TCGZ1 | 4.52 | 13.08 | 31.08 | 43.10 | 40.38 | 30.61 | 19.92 | 11.02 | 4.11 | 2.18 |
| TCGZ10 | 3.80 | 4.99 | 27.23 | 30.16 | 40.71 | 38.90 | 23.16 | 20.96 | 5.11 | 4.99 |
| TCGZ3 | 4.43 | 12.37 | 30.86 | 42.62 | 40.40 | 31.10 | 20.10 | 11.55 | 4.20 | 2.36 |
| TCGZ4 | 4.37 | 5.87 | 30.64 | 35.46 | 40.31 | 38.14 | 20.39 | 16.87 | 4.29 | 3.66 |
| TCGZ5 | 4.42 | 13.19 | 30.72 | 42.70 | 40.37 | 30.43 | 20.27 | 11.35 | 4.23 | 2.34 |
| TCGZ6 | 4.49 | 11.27 | 30.83 | 41.27 | 40.49 | 32.33 | 20.00 | 12.48 | 4.17 | 2.65 |
| TCLZ1 | 4.36 | 5.98 | 30.00 | 35.75 | 40.28 | 37.66 | 20.86 | 16.82 | 4.49 | 3.79 |
| TCLZ2 | 4.27 | 5.25 | 29.83 | 33.25 | 40.64 | 39.16 | 20.80 | 18.28 | 4.47 | 4.06 |
| TCLZ3 | 4.25 | 7.62 | 29.79 | 37.41 | 40.13 | 35.78 | 21.23 | 15.66 | 4.60 | 3.52 |
| TCLZ4 | 4.39 | 5.65 | 30.18 | 33.74 | 40.40 | 38.78 | 20.65 | 17.86 | 4.38 | 3.97 |
| TCLZ5 | 4.48 | 9.48 | 30.76 | 39.06 | 40.36 | 34.31 | 20.11 | 14.07 | 4.29 | 3.08 |
| TCQH1 | 4.37 | 7.64 | 30.65 | 38.67 | 40.38 | 36.00 | 20.27 | 14.43 | 4.33 | 3.26 |
| TCQH10 | 4.52 | 13.19 | 31.19 | 43.18 | 40.25 | 30.42 | 19.91 | 10.99 | 4.13 | 2.22 |
| TCQH11 | 4.44 | 13.09 | 31.13 | 43.18 | 40.41 | 30.48 | 19.83 | 11.01 | 4.19 | 2.24 |
| TCQH5 | 4.25 | 8.71 | 28.92 | 34.55 | 39.87 | 35.06 | 21.75 | 17.27 | 5.21 | 4.41 |
| TCQH8 | 4.48 | 12.95 | 30.96 | 43.08 | 40.38 | 30.56 | 19.98 | 11.13 | 4.20 | 2.28 |
| TCQH9 | 4.45 | 12.89 | 31.13 | 43.12 | 40.27 | 30.56 | 19.98 | 11.17 | 4.17 | 2.26 |
| TCSN1 | 4.49 | 13.35 | 30.98 | 43.24 | 40.42 | 30.53 | 19.99 | 10.78 | 4.12 | 2.10 |
| TCSN3 | 4.53 | 13.50 | 31.19 | 43.25 | 40.37 | 30.22 | 19.76 | 10.83 | 4.14 | 2.20 |
| TCSN4 | 4.48 | 13.27 | 30.95 | 43.20 | 40.24 | 30.28 | 20.06 | 11.00 | 4.26 | 2.26 |
| TCSN5 | 4.29 | 9.14 | 29.51 | 38.07 | 40.39 | 34.29 | 21.26 | 15.14 | 4.55 | 3.37 |
| TCSN6 | 4.39 | 9.78 | 30.68 | 40.54 | 40.17 | 33.43 | 20.41 | 13.39 | 4.35 | 2.85 |
| TCSN7 | 4.59 | 13.24 | 31.52 | 43.76 | 40.47 | 30.66 | 19.52 | 10.43 | 3.90 | 1.91 |
| TCSN8 | 4.49 | 13.12 | 31.58 | 43.81 | 40.56 | 30.72 | 19.48 | 10.48 | 3.87 | 1.87 |
| TCSN9 | 4.37 | 11.22 | 30.91 | 42.22 | 40.55 | 32.37 | 20.06 | 11.87 | 4.11 | 2.32 |
| YNLC1 | 4.49 | 14.40 | 30.94 | 43.20 | 40.61 | 29.98 | 19.87 | 10.48 | 4.09 | 1.94 |
| YNLC2 | 4.51 | 14.80 | 31.32 | 44.11 | 40.77 | 29.70 | 19.48 | 9.70 | 3.91 | 1.69 |
| YNLC3 | 4.49 | 14.73 | 31.06 | 43.72 | 40.45 | 29.62 | 19.95 | 10.17 | 4.04 | 1.76 |
| YNLC4 | 4.52 | 14.92 | 30.92 | 43.26 | 40.48 | 29.52 | 19.90 | 10.29 | 4.18 | 2.01 |
| YNLC5 | 4.48 | 14.41 | 30.91 | 43.67 | 40.68 | 29.97 | 19.89 | 10.18 | 4.05 | 1.77 |
| YNLC6 | 4.41 | 14.69 | 31.07 | 43.68 | 40.69 | 29.78 | 19.83 | 10.08 | 4.01 | 1.77 |
| YNLC7 | 4.00 | 7.63 | 27.88 | 35.22 | 41.01 | 36.75 | 22.32 | 16.79 | 4.79 | 3.61 |
| YNLC8 | 4.43 | 14.80 | 31.43 | 44.11 | 40.70 | 29.48 | 19.53 | 9.87 | 3.92 | 1.73 |


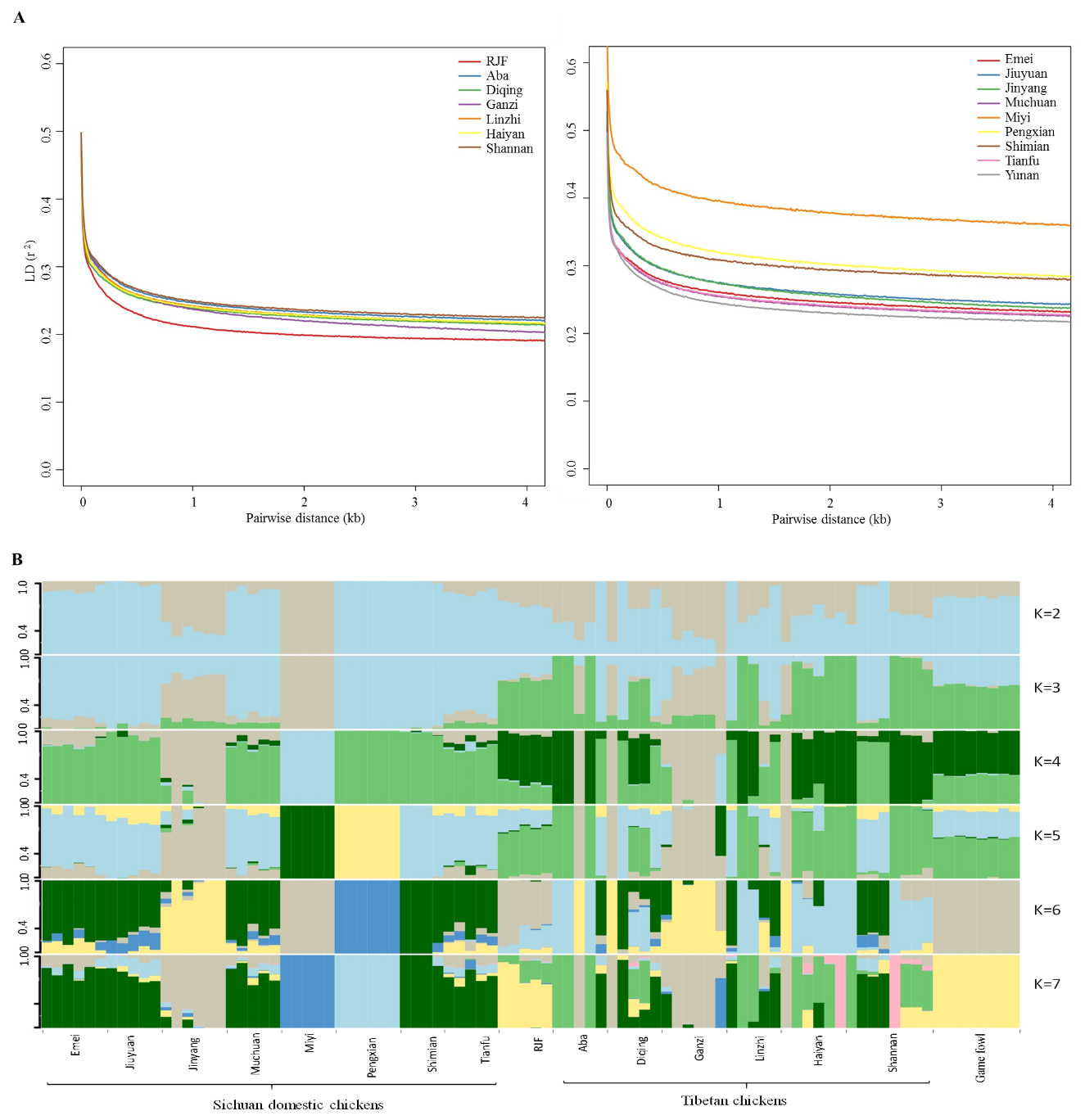


**Figure S7** LD decay and population structures with numbers of ancestral clusters (K) from 2 to 7. (A) Each color represents one ancestral cluster, and each vertical bar represents one chicken. (B) The length of each colored segment represents the corresponding ancestry attribution.

**Table S4** Symmetric four-taxon ABBA/BABA tests for all combinations of the 15 chicken populations (9 domestic chicken breeds and 6 Tibetan chicken populations) and the red jungle fowl. A naïve binomial test for a difference in the rates of ABBA and BABA counts is incorrect because of linkage disequilibrium. Shading is used to highlight P-values greater than 0.001. The analyses below are based on computing the D-statistic (P1, P2, P3, Japanese quail) using P3= red jungle fowls. P3 is the population from which we queried derived alleles in P1 and P2. A positive D means P3 is closer to P2 than to P1 (ABBA excess), and a negative D mean P3 is close to P1 than to P2 (BABA excess).

| P1 | P2 | P3 | Outgroup (P4) | *P* value | Number  of ABBA  sites | Number  of BABA  sites | D=(ABBA-BABA)  /(ABBA+BABA) |
| --- | --- | --- | --- | --- | --- | --- | --- |
| Game fowl | Jinyang | RJF | Japanese Quail | 0.983246 | 47190 | 47186 | 4.24E-05 |
| Game fowl | Ganzi | RJF | Japanese Quail | 0.065038 | 47188 | 47465 | -0.00293 |
| Game fowl | Pengxian | RJF | Japanese Quail | 0.024322 | 46740 | 47114 | -0.00398 |
| Game fowl | Miyi | RJF | Japanese Quail | 0.002382 | 47136 | 47665 | -0.00558 |
| Game fowl | Jiuyuan | RJF | Japanese Quail | 0.000328 | 46527 | 47098 | -0.0061 |
| Game fowl | Muchuan | RJF | Japanese Quail | 1.25E-10 | 46432 | 47463 | -0.01098 |
| Game fowl | Emei | RJF | Japanese Quail | 8.41E-11 | 46238 | 47277 | -0.01111 |
| Game fowl | Tianfu | RJF | Japanese Quail | 3.25E-11 | 46531 | 47569 | -0.01103 |
| Game fowl | Shannan | RJF | Japanese Quail | 2.19E-12 | 46612 | 47674 | -0.01126 |
| Game fowl | Haiyan | RJF | Japanese Quail | 1.51E-19 | 46711 | 48218 | -0.01588 |
| Game fowl | Shimian | RJF | Japanese Quail | 1.54E-22 | 45998 | 47775 | -0.01895 |
| Game fowl | Aba | RJF | Japanese Quail | 5.36E-24 | 46608 | 48264 | -0.01746 |
| Game fowl | Diqing | RJF | Japanese Quail | 3.81E-25 | 46407 | 47856 | -0.01537 |
| Game fowl | Linzhi | RJF | Japanese Quail | 7.76E-30 | 46254 | 48040 | -0.01894 |
| Shannan | Emei | RJF | Japanese Quail | 0.870518 | 46056 | 46034 | 0.000239 |
| Shannan | Tianfu | RJF | Japanese Quail | 0.848526 | 46321 | 46297 | 0.000259 |
| Shannan | Muchuan | RJF | Japanese Quail | 0.800268 | 46282 | 46252 | 0.000324 |
| Shannan | Miyi | RJF | Japanese Quail | 0.001027 | 46927 | 46396 | 0.00569 |
| Shannan | Jiuyuan | RJF | Japanese Quail | 0.000318 | 46471 | 45980 | 0.005311 |
| Shannan | Diqing | RJF | Japanese Quail | 3.72E-05 | 45957 | 46345 | -0.0042 |
| Shannan | Haiyan | RJF | Japanese Quail | 2.97E-06 | 45144 | 45590 | -0.00492 |
| Shannan | Pengxian | RJF | Japanese Quail | 1.26E-06 | 46605 | 45917 | 0.007436 |
| Shannan | Shimian | RJF | Japanese Quail | 8.79E-07 | 45695 | 46410 | -0.00776 |
| Shannan | Aba | RJF | Japanese Quail | 5.25E-09 | 45454 | 46048 | -0.00649 |
| Shannan | Game fowl | RJF | Japanese Quail | 2.19E-12 | 47674 | 46612 | 0.011264 |
| Shannan | Ganzi | RJF | Japanese Quail | 7.32E-14 | 46757 | 45972 | 0.008466 |
| Shannan | Linzhi | RJF | Japanese Quail | 1.79E-14 | 45220 | 45945 | -0.00795 |
| Shannan | Jinyang | RJF | Japanese Quail | 1.01E-17 | 46932 | 45868 | 0.011466 |
| Haiyan | Diqing | RJF | Japanese Quail | 0.580263 | 46234 | 46175 | 0.000638 |
| Haiyan | Aba | RJF | Japanese Quail | 0.178625 | 45045 | 45193 | -0.00164 |
| Haiyan | Shimian | RJF | Japanese Quail | 0.081334 | 46530 | 46800 | -0.00289 |
| Haiyan | Linzhi | RJF | Japanese Quail | 0.013855 | 45614 | 45893 | -0.00305 |
| Haiyan | Emei | RJF | Japanese Quail | 0.001627 | 46871 | 46403 | 0.005017 |
| Haiyan | Tianfu | RJF | Japanese Quail | 0.001247 | 47089 | 46618 | 0.005026 |
| Haiyan | Muchuan | RJF | Japanese Quail | 0.00072 | 47053 | 46576 | 0.005095 |
| Haiyan | Shannan | RJF | Japanese Quail | 2.97E-06 | 45590 | 45144 | 0.004915 |
| Haiyan | Miyi | RJF | Japanese Quail | 6.67E-09 | 46812 | 45834 | 0.010556 |
| Haiyan | Jiuyuan | RJF | Japanese Quail | 6.7E-10 | 47454 | 46517 | 0.009971 |
| Haiyan | Pengxian | RJF | Japanese Quail | 3.68E-13 | 47650 | 46517 | 0.012032 |
| Haiyan | Game fowl | RJF | Japanese Quail | 1.51E-19 | 48218 | 46711 | 0.015875 |
| Haiyan | Ganzi | RJF | Japanese Quail | 3.42E-26 | 46538 | 45307 | 0.013403 |
| Haiyan | Jinyang | RJF | Japanese Quail | 1.27E-29 | 46862 | 45351 | 0.016386 |
| Linzhi | Shimian | RJF | Japanese Quail | 0.945786 | 45279 | 45270 | 9.94E-05 |
| Linzhi | Aba | RJF | Japanese Quail | 0.226663 | 45268 | 45137 | 0.001449 |
| Linzhi | Haiyan | RJF | Japanese Quail | 0.013855 | 45893 | 45614 | 0.003049 |
| Linzhi | Diqing | RJF | Japanese Quail | 0.000635 | 45755 | 45418 | 0.003696 |
| Linzhi | Emei | RJF | Japanese Quail | 2.05E-09 | 45647 | 44900 | 0.00825 |
| Linzhi | Tianfu | RJF | Japanese Quail | 6.09E-10 | 45999 | 45250 | 0.008208 |
| Linzhi | Muchuan | RJF | Japanese Quail | 3.5E-11 | 45945 | 45189 | 0.008295 |
| Linzhi | Shannan | RJF | Japanese Quail | 1.79E-14 | 45945 | 45220 | 0.007953 |
| Linzhi | Miyi | RJF | Japanese Quail | 6.65E-15 | 46778 | 45522 | 0.013608 |
| Linzhi | Jiuyuan | RJF | Japanese Quail | 7.42E-23 | 46134 | 44918 | 0.013355 |
| Linzhi | Pengxian | RJF | Japanese Quail | 8.07E-25 | 46255 | 44843 | 0.0155 |
| Linzhi | Game fowl | RJF | Japanese Quail | 7.76E-30 | 48040 | 46254 | 0.018941 |
| Linzhi | Ganzi | RJF | Japanese Quail | 1.38E-39 | 46617 | 45108 | 0.016451 |
| Linzhi | Jinyang | RJF | Japanese Quail | 2.34E-43 | 46696 | 44907 | 0.01953 |
| Ganzi | Pengxian | RJF | Japanese Quail | 0.504134 | 45953 | 46050 | -0.00105 |
| Ganzi | Game fowl | RJF | Japanese Quail | 0.065038 | 47465 | 47188 | 0.002926 |
| Ganzi | Miyi | RJF | Japanese Quail | 0.039303 | 42616 | 42870 | -0.00297 |
| Ganzi | Jiuyuan | RJF | Japanese Quail | 0.016711 | 45765 | 46058 | -0.00319 |
| Ganzi | Jinyang | RJF | Japanese Quail | 0.006668 | 43815 | 43535 | 0.003205 |
| Ganzi | Muchuan | RJF | Japanese Quail | 2.67E-08 | 45491 | 46245 | -0.00822 |
| Ganzi | Tianfu | RJF | Japanese Quail | 7E-09 | 45536 | 46296 | -0.00828 |
| Ganzi | Emei | RJF | Japanese Quail | 1.06E-09 | 44987 | 45750 | -0.00841 |
| Ganzi | Shannan | RJF | Japanese Quail | 7.32E-14 | 45972 | 46757 | -0.00847 |
| Ganzi | Shimian | RJF | Japanese Quail | 3.69E-25 | 45088 | 46588 | -0.01636 |
| Ganzi | Haiyan | RJF | Japanese Quail | 3.42E-26 | 45307 | 46538 | -0.0134 |
| Ganzi | Aba | RJF | Japanese Quail | 2.04E-29 | 45076 | 46454 | -0.01506 |
| Ganzi | Diqing | RJF | Japanese Quail | 6.17E-34 | 44915 | 46088 | -0.01289 |
| Ganzi | Linzhi | RJF | Japanese Quail | 1.38E-39 | 45108 | 46617 | -0.01645 |
| Diqing | Haiyan | RJF | Japanese Quail | 0.580263 | 46175 | 46234 | -0.00064 |
| Diqing | Aba | RJF | Japanese Quail | 0.037894 | 45799 | 46006 | -0.00225 |
| Diqing | Shimian | RJF | Japanese Quail | 0.017454 | 45316 | 45643 | -0.0036 |
| Diqing | Emei | RJF | Japanese Quail | 0.000667 | 45646 | 45237 | 0.0045 |
| Diqing | Linzhi | RJF | Japanese Quail | 0.000635 | 45418 | 45755 | -0.0037 |
| Diqing | Muchuan | RJF | Japanese Quail | 7.28E-05 | 45840 | 45422 | 0.00458 |
| Diqing | Tianfu | RJF | Japanese Quail | 3.82E-05 | 45924 | 45512 | 0.004506 |
| Diqing | Shannan | RJF | Japanese Quail | 3.72E-05 | 46345 | 45957 | 0.004204 |
| Diqing | Miyi | RJF | Japanese Quail | 5.38E-09 | 46354 | 45435 | 0.010012 |
| Diqing | Jiuyuan | RJF | Japanese Quail | 7.15E-16 | 46070 | 45192 | 0.009621 |
| Diqing | Pengxian | RJF | Japanese Quail | 1.49E-17 | 46184 | 45108 | 0.011786 |
| Diqing | Game fowl | RJF | Japanese Quail | 3.81E-25 | 47856 | 46407 | 0.015372 |
| Diqing | Ganzi | RJF | Japanese Quail | 6.17E-34 | 46088 | 44915 | 0.01289 |
| Diqing | Jinyang | RJF | Japanese Quail | 3.11E-35 | 46308 | 44855 | 0.015938 |
| Aba | Shimian | RJF | Japanese Quail | 0.4413 | 46293 | 46414 | -0.00131 |
| Aba | Linzhi | RJF | Japanese Quail | 0.226663 | 45137 | 45268 | -0.00145 |
| Aba | Haiyan | RJF | Japanese Quail | 0.178625 | 45193 | 45045 | 0.00164 |
| Aba | Diqing | RJF | Japanese Quail | 0.037894 | 46006 | 45799 | 0.002255 |
| Aba | Emei | RJF | Japanese Quail | 1.59E-05 | 46600 | 45984 | 0.006653 |
| Aba | Tianfu | RJF | Japanese Quail | 3.67E-06 | 46799 | 46181 | 0.006647 |
| Aba | Muchuan | RJF | Japanese Quail | 1.54E-06 | 46743 | 46118 | 0.00673 |
| Aba | Shannan | RJF | Japanese Quail | 5.25E-09 | 46048 | 45454 | 0.006492 |
| Aba | Miyi | RJF | Japanese Quail | 2.67E-10 | 46793 | 45667 | 0.012178 |
| Aba | Jiuyuan | RJF | Japanese Quail | 6.29E-14 | 47199 | 46114 | 0.011628 |
| Aba | Pengxian | RJF | Japanese Quail | 1.46E-16 | 47352 | 46071 | 0.013712 |
| Aba | Game fowl | RJF | Japanese Quail | 5.36E-24 | 48264 | 46608 | 0.017455 |
| Aba | Ganzi | RJF | Japanese Quail | 2.04E-29 | 46454 | 45076 | 0.015055 |
| Aba | Jinyang | RJF | Japanese Quail | 6.73E-33 | 46814 | 45155 | 0.018039 |
| Tianfu | Emei | RJF | Japanese Quail | 0.985639 | 44746 | 44748 | -2.2E-05 |
| Tianfu | Muchuan | RJF | Japanese Quail | 0.950563 | 44060 | 44053 | 7.94E-05 |
| Tianfu | Shannan | RJF | Japanese Quail | 0.848526 | 46297 | 46321 | -0.00026 |
| Tianfu | Miyi | RJF | Japanese Quail | 0.003603 | 46064 | 45558 | 0.005523 |
| Tianfu | Haiyan | RJF | Japanese Quail | 0.001247 | 46618 | 47089 | -0.00503 |
| Tianfu | Diqing | RJF | Japanese Quail | 3.82E-05 | 45512 | 45924 | -0.00451 |
| Tianfu | Jiuyuan | RJF | Japanese Quail | 2.73E-05 | 44796 | 44329 | 0.00524 |
| Tianfu | Pengxian | RJF | Japanese Quail | 5.09E-06 | 45084 | 44421 | 0.007407 |
| Tianfu | Aba | RJF | Japanese Quail | 3.67E-06 | 46181 | 46799 | -0.00665 |
| Tianfu | Shimian | RJF | Japanese Quail | 5.94E-07 | 44403 | 45142 | -0.00825 |
| Tianfu | Ganzi | RJF | Japanese Quail | 7E-09 | 46296 | 45536 | 0.008276 |
| Tianfu | Linzhi | RJF | Japanese Quail | 6.09E-10 | 45250 | 45999 | -0.00821 |
| Tianfu | Game fowl | RJF | Japanese Quail | 3.25E-11 | 47569 | 46531 | 0.011031 |
| Tianfu | Jinyang | RJF | Japanese Quail | 1.01E-13 | 46247 | 45206 | 0.011383 |
| Shimian | Linzhi | RJF | Japanese Quail | 0.945786 | 45270 | 45279 | -9.9E-05 |
| Shimian | Aba | RJF | Japanese Quail | 0.4413 | 46414 | 46293 | 0.001305 |
| Shimian | Haiyan | RJF | Japanese Quail | 0.081334 | 46800 | 46530 | 0.002893 |
| Shimian | Diqing | RJF | Japanese Quail | 0.017454 | 45643 | 45316 | 0.003595 |
| Shimian | Muchuan | RJF | Japanese Quail | 1.2E-06 | 45033 | 44287 | 0.008352 |
| Shimian | Shannan | RJF | Japanese Quail | 8.79E-07 | 46410 | 45695 | 0.007763 |
| Shimian | Tianfu | RJF | Japanese Quail | 5.94E-07 | 45142 | 44403 | 0.008253 |
| Shimian | Emei | RJF | Japanese Quail | 4.83E-07 | 44625 | 43888 | 0.008326 |
| Shimian | Miyi | RJF | Japanese Quail | 1.37E-11 | 46762 | 45515 | 0.013514 |
| Shimian | Jiuyuan | RJF | Japanese Quail | 1.32E-15 | 45047 | 43841 | 0.013568 |
| Shimian | Pengxian | RJF | Japanese Quail | 1.99E-19 | 45009 | 43607 | 0.015821 |
| Shimian | Game fowl | RJF | Japanese Quail | 1.54E-22 | 47775 | 45998 | 0.01895 |
| Shimian | Ganzi | RJF | Japanese Quail | 3.69E-25 | 46588 | 45088 | 0.016362 |
| Shimian | Jinyang | RJF | Japanese Quail | 2.3E-27 | 46665 | 44885 | 0.019443 |
| Pengxian | Ganzi | RJF | Japanese Quail | 0.504134 | 46050 | 45953 | 0.001054 |
| Pengxian | Miyi | RJF | Japanese Quail | 0.390341 | 46292 | 46448 | -0.00168 |
| Pengxian | Jiuyuan | RJF | Japanese Quail | 0.14902 | 44101 | 44297 | -0.00222 |
| Pengxian | Game fowl | RJF | Japanese Quail | 0.024322 | 47114 | 46740 | 0.003985 |
| Pengxian | Jinyang | RJF | Japanese Quail | 0.019965 | 46018 | 45641 | 0.004113 |
| Pengxian | Tianfu | RJF | Japanese Quail | 5.09E-06 | 44421 | 45084 | -0.00741 |
| Pengxian | Muchuan | RJF | Japanese Quail | 2.56E-06 | 44345 | 45002 | -0.00735 |
| Pengxian | Shannan | RJF | Japanese Quail | 1.26E-06 | 45917 | 46605 | -0.00744 |
| Pengxian | Emei | RJF | Japanese Quail | 4.28E-07 | 43892 | 44557 | -0.00752 |
| Pengxian | Haiyan | RJF | Japanese Quail | 3.68E-13 | 46517 | 47650 | -0.01203 |
| Pengxian | Aba | RJF | Japanese Quail | 1.46E-16 | 46071 | 47352 | -0.01371 |
| Pengxian | Diqing | RJF | Japanese Quail | 1.49E-17 | 45108 | 46184 | -0.01179 |
| Pengxian | Shimian | RJF | Japanese Quail | 1.99E-19 | 43607 | 45009 | -0.01582 |
| Pengxian | Linzhi | RJF | Japanese Quail | 8.07E-25 | 44843 | 46255 | -0.0155 |
| Miyi | Jiuyuan | RJF | Japanese Quail | 0.808005 | 46152 | 46191 | -0.00042 |
| Miyi | Pengxian | RJF | Japanese Quail | 0.390341 | 46448 | 46292 | 0.001682 |
| Miyi | Ganzi | RJF | Japanese Quail | 0.039303 | 42870 | 42616 | 0.002971 |
| Miyi | Muchuan | RJF | Japanese Quail | 0.005469 | 45757 | 46258 | -0.00544 |
| Miyi | Tianfu | RJF | Japanese Quail | 0.003603 | 45558 | 46064 | -0.00552 |
| Miyi | Game fowl | RJF | Japanese Quail | 0.002382 | 47665 | 47136 | 0.00558 |
| Miyi | Emei | RJF | Japanese Quail | 0.001935 | 45287 | 45795 | -0.00558 |
| Miyi | Shannan | RJF | Japanese Quail | 0.001027 | 46396 | 46927 | -0.00569 |
| Miyi | Jinyang | RJF | Japanese Quail | 0.000133 | 43521 | 42988 | 0.006161 |
| Miyi | Haiyan | RJF | Japanese Quail | 6.67E-09 | 45834 | 46812 | -0.01056 |
| Miyi | Diqing | RJF | Japanese Quail | 5.38E-09 | 45435 | 46354 | -0.01001 |
| Miyi | Aba | RJF | Japanese Quail | 2.67E-10 | 45667 | 46793 | -0.01218 |
| Miyi | Shimian | RJF | Japanese Quail | 1.37E-11 | 45515 | 46762 | -0.01351 |
| Miyi | Linzhi | RJF | Japanese Quail | 6.65E-15 | 45522 | 46778 | -0.01361 |
| Muchuan | Tianfu | RJF | Japanese Quail | 0.950563 | 44053 | 44060 | -7.9E-05 |
| Muchuan | Emei | RJF | Japanese Quail | 0.945786 | 44623 | 44632 | -0.0001 |
| Muchuan | Shannan | RJF | Japanese Quail | 0.800268 | 46252 | 46282 | -0.00032 |
| Muchuan | Miyi | RJF | Japanese Quail | 0.005469 | 46258 | 45757 | 0.005445 |
| Muchuan | Haiyan | RJF | Japanese Quail | 0.00072 | 46576 | 47053 | -0.00509 |
| Muchuan | Jiuyuan | RJF | Japanese Quail | 0.000181 | 44730 | 44270 | 0.005169 |
| Muchuan | Diqing | RJF | Japanese Quail | 7.28E-05 | 45422 | 45840 | -0.00458 |
| Muchuan | Pengxian | RJF | Japanese Quail | 2.56E-06 | 45002 | 44345 | 0.007353 |
| Muchuan | Aba | RJF | Japanese Quail | 1.54E-06 | 46118 | 46743 | -0.00673 |
| Muchuan | Shimian | RJF | Japanese Quail | 1.2E-06 | 44287 | 45033 | -0.00835 |
| Muchuan | Ganzi | RJF | Japanese Quail | 2.67E-08 | 46245 | 45491 | 0.008219 |
| Muchuan | Game fowl | RJF | Japanese Quail | 1.25E-10 | 47463 | 46432 | 0.01098 |
| Muchuan | Linzhi | RJF | Japanese Quail | 3.5E-11 | 45189 | 45945 | -0.0083 |
| Muchuan | Jinyang | RJF | Japanese Quail | 8.61E-12 | 46192 | 45158 | 0.011319 |
| Jinyang | Game fowl | RJF | Japanese Quail | 0.983246 | 47186 | 47190 | -4.2E-05 |
| Jinyang | Pengxian | RJF | Japanese Quail | 0.019965 | 45641 | 46018 | -0.00411 |
| Jinyang | Ganzi | RJF | Japanese Quail | 0.006668 | 43535 | 43815 | -0.00321 |
| Jinyang | Miyi | RJF | Japanese Quail | 0.000133 | 42988 | 43521 | -0.00616 |
| Jinyang | Jiuyuan | RJF | Japanese Quail | 9.58E-05 | 45455 | 46029 | -0.00627 |
| Jinyang | Muchuan | RJF | Japanese Quail | 8.61E-12 | 45158 | 46192 | -0.01132 |
| Jinyang | Tianfu | RJF | Japanese Quail | 1.01E-13 | 45206 | 46247 | -0.01138 |
| Jinyang | Emei | RJF | Japanese Quail | 3.91E-14 | 44544 | 45587 | -0.01157 |
| Jinyang | Shannan | RJF | Japanese Quail | 1.01E-17 | 45868 | 46932 | -0.01147 |
| Jinyang | Shimian | RJF | Japanese Quail | 2.3E-27 | 44885 | 46665 | -0.01944 |
| Jinyang | Haiyan | RJF | Japanese Quail | 1.27E-29 | 45351 | 46862 | -0.01639 |
| Jinyang | Aba | RJF | Japanese Quail | 6.73E-33 | 45155 | 46814 | -0.01804 |
| Jinyang | Diqing | RJF | Japanese Quail | 3.11E-35 | 44855 | 46308 | -0.01594 |
| Jinyang | Linzhi | RJF | Japanese Quail | 2.34E-43 | 44907 | 46696 | -0.01953 |
| Jiuyuan | Miyi | RJF | Japanese Quail | 0.808005 | 46191 | 46152 | 0.000422 |
| Jiuyuan | Pengxian | RJF | Japanese Quail | 0.14902 | 44297 | 44101 | 0.002217 |
| Jiuyuan | Ganzi | RJF | Japanese Quail | 0.016711 | 46058 | 45765 | 0.003191 |
| Jiuyuan | Game fowl | RJF | Japanese Quail | 0.000328 | 47098 | 46527 | 0.006099 |
| Jiuyuan | Shannan | RJF | Japanese Quail | 0.000318 | 45980 | 46471 | -0.00531 |
| Jiuyuan | Muchuan | RJF | Japanese Quail | 0.000181 | 44270 | 44730 | -0.00517 |
| Jiuyuan | Jinyang | RJF | Japanese Quail | 9.58E-05 | 46029 | 45455 | 0.006274 |
| Jiuyuan | Emei | RJF | Japanese Quail | 6.98E-05 | 43902 | 44371 | -0.00531 |
| Jiuyuan | Tianfu | RJF | Japanese Quail | 2.73E-05 | 44329 | 44796 | -0.00524 |
| Jiuyuan | Haiyan | RJF | Japanese Quail | 6.7E-10 | 46517 | 47454 | -0.00997 |
| Jiuyuan | Aba | RJF | Japanese Quail | 6.29E-14 | 46114 | 47199 | -0.01163 |
| Jiuyuan | Shimian | RJF | Japanese Quail | 1.32E-15 | 43841 | 45047 | -0.01357 |
| Jiuyuan | Diqing | RJF | Japanese Quail | 7.15E-16 | 45192 | 46070 | -0.00962 |
| Jiuyuan | Linzhi | RJF | Japanese Quail | 7.42E-23 | 44918 | 46134 | -0.01336 |
| Emei | Tianfu | RJF | Japanese Quail | 0.985639 | 44748 | 44746 | 2.23E-05 |
| Emei | Muchuan | RJF | Japanese Quail | 0.945786 | 44632 | 44623 | 0.000101 |
| Emei | Shannan | RJF | Japanese Quail | 0.870518 | 46034 | 46056 | -0.00024 |
| Emei | Miyi | RJF | Japanese Quail | 0.001935 | 45795 | 45287 | 0.005577 |
| Emei | Haiyan | RJF | Japanese Quail | 0.001627 | 46403 | 46871 | -0.00502 |
| Emei | Diqing | RJF | Japanese Quail | 0.000667 | 45237 | 45646 | -0.0045 |
| Emei | Jiuyuan | RJF | Japanese Quail | 6.98E-05 | 44371 | 43902 | 0.005313 |
| Emei | Aba | RJF | Japanese Quail | 1.59E-05 | 45984 | 46600 | -0.00665 |
| Emei | Shimian | RJF | Japanese Quail | 4.83E-07 | 43888 | 44625 | -0.00833 |
| Emei | Pengxian | RJF | Japanese Quail | 4.28E-07 | 44557 | 43892 | 0.007518 |
| Emei | Linzhi | RJF | Japanese Quail | 2.05E-09 | 44900 | 45647 | -0.00825 |
| Emei | Ganzi | RJF | Japanese Quail | 1.06E-09 | 45750 | 44987 | 0.008409 |
| Emei | Game fowl | RJF | Japanese Quail | 8.41E-11 | 47277 | 46238 | 0.011111 |
| Emei | Jinyang | RJF | Japanese Quail | 3.91E-14 | 45587 | 44544 | 0.011572 |

**Table S5** Tracts of identity by descent (IBD) between each pair of chicken populations.

| Pop1 | Pop2 | Average IBD length (bp) | Media IBD length (bp) | Total IBD length (bp) | Number of IBD |
| --- | --- | --- | --- | --- | --- |
| RJF | Shimian | 31885.59 | 16756 | 44129655 | 1384 |
| RJF | Miyi | 40307.63 | 21913.5 | 58929748 | 1462 |
| RJF | Linzhi | 34818.60 | 17825 | 62360111 | 1791 |
| RJF | Emei | 37784.07 | 18867.5 | 82444835 | 2182 |
| RJF | Aba | 38063.38 | 19936.5 | 86708390 | 2278 |
| RJF | Jiuyuan | 42746.40 | 22458 | 99556368 | 2329 |
| RJF | Muchuan | 41380.45 | 20880 | 102623521 | 2480 |
| RJF | Jinyang | 43019.82 | 22653 | 110259804 | 2563 |
| RJF | Haiyan | 35373.54 | 20366.5 | 96216042 | 2720 |
| RJF | Ganzi | 44531.37 | 21808 | 123975326 | 2784 |
| RJF | Pengxian | 45312.35 | 23563 | 136344848 | 3009 |
| RJF | Diqing | 41014.49 | 20905 | 127432020 | 3107 |
| RJF | Tianfu | 42851.85 | 22015 | 136911656 | 3195 |
| RJF | Shannan | 40118.53 | 21172 | 191846830 | 4782 |
| RJF | Game fowl | 46720.26 | 23445.5 | 637357814 | 13642 |
| Emei | RJF | 37784.07 | 18867.5 | 82444835 | 2182 |
| Emei | Haiyan | 34409.73 | 19099 | 152848018 | 4442 |
| Emei | Miyi | 40505.64 | 20775 | 180695639 | 4461 |
| Emei | Aba | 33514.10 | 18361 | 155002721 | 4625 |
| Emei | Linzhi | 30166.15 | 17256 | 198794939 | 6590 |
| Emei | Shimian | 28477.80 | 16336 | 188095863 | 6605 |
| Emei | Game fowl | 39336.43 | 19946 | 269257831 | 6845 |
| Emei | Ganzi | 39251.82 | 20228 | 295605431 | 7531 |
| Emei | Jinyang | 36609.19 | 20103 | 317657915 | 8677 |
| Emei | Diqing | 35019.45 | 19221 | 314369585 | 8977 |
| Emei | Jiuyuan | 41284.68 | 20985 | 430392793 | 10425 |
| Emei | Muchuan | 35880.64 | 19641 | 378253757 | 10542 |
| Emei | Shannan | 35796.59 | 19602 | 381949585 | 10670 |
| Emei | Tianfu | 40164.85 | 21805 | 479608459 | 11941 |
| Emei | Pengxian | 43331.20 | 22180 | 554856030 | 12805 |
| Jiuyuan | RJF | 42746.40 | 22458 | 99556368 | 2329 |
| Jiuyuan | Miyi | 46161.44 | 25054.5 | 149286089 | 3234 |
| Jiuyuan | Haiyan | 45038.68 | 20891.5 | 180965429 | 4018 |
| Jiuyuan | Aba | 40012.01 | 22055 | 187736358 | 4692 |
| Jiuyuan | Linzhi | 37379.15 | 19198 | 206893591 | 5535 |
| Jiuyuan | Shimian | 32847.29 | 18059 | 184503208 | 5617 |
| Jiuyuan | Ganzi | 46223.15 | 23446.5 | 277893560 | 6012 |
| Jiuyuan | Jinyang | 44747.28 | 23965 | 300567512 | 6717 |
| Jiuyuan | Game fowl | 43435.56 | 22247 | 335626550 | 7727 |
| Jiuyuan | Diqing | 39038.52 | 21276 | 327103720 | 8379 |
| Jiuyuan | Muchuan | 44018.12 | 22912 | 455279386 | 10343 |
| Jiuyuan | Emei | 41284.68 | 20985 | 430392793 | 10425 |
| Jiuyuan | Shannan | 41306.99 | 21750 | 439712930 | 10645 |
| Jiuyuan | Tianfu | 46099.26 | 25113 | 559137963 | 12129 |
| Jiuyuan | Pengxian | 53219.51 | 26109 | 720379318 | 13536 |
| Jinyang | RJF | 43019.82 | 22653 | 110259804 | 2563 |
| Jinyang | Shimian | 32868.01 | 17920.5 | 122794875 | 3736 |
| Jinyang | Aba | 36973.24 | 20188.5 | 189968502 | 5138 |
| Jinyang | Linzhi | 36593.09 | 19568 | 233207742 | 6373 |
| Jinyang | Jiuyuan | 44747.28 | 23965 | 300567512 | 6717 |
| Jinyang | Haiyan | 36281.10 | 20168 | 271455173 | 7482 |
| Jinyang | Game fowl | 43609.40 | 21956 | 335617964 | 7696 |
| Jinyang | Muchuan | 41931.83 | 21479.5 | 344260334 | 8210 |
| Jinyang | Emei | 36609.19 | 20103 | 317657915 | 8677 |
| Jinyang | Pengxian | 48152.45 | 25457.5 | 425186137 | 8830 |
| Jinyang | Shannan | 41740.78 | 22231 | 410520565 | 9835 |
| Jinyang | Tianfu | 44435.16 | 23204 | 442574181 | 9960 |
| Jinyang | Diqing | 42288.50 | 21851.5 | 499088864 | 11802 |
| Jinyang | Miyi | 46214.89 | 23644 | 551574706 | 11935 |
| Jinyang | Ganzi | 43104.62 | 22892 | 877523911 | 20358 |
| Muchuan | RJF | 41380.45 | 20880 | 102623521 | 2480 |
| Muchuan | Miyi | 43005.05 | 23062 | 188921187 | 4393 |
| Muchuan | Haiyan | 37848.27 | 20409 | 194767221 | 5146 |
| Muchuan | Aba | 36978.20 | 20867.5 | 249380972 | 6744 |
| Muchuan | Shimian | 30800.21 | 17902 | 224625934 | 7293 |
| Muchuan | Linzhi | 31905.49 | 18357 | 235303023 | 7375 |
| Muchuan | Ganzi | 41891.38 | 22190.5 | 327925735 | 7828 |
| Muchuan | Jinyang | 41931.83 | 21479.5 | 344260334 | 8210 |
| Muchuan | Game fowl | 40555.55 | 20911 | 337706083 | 8327 |
| Muchuan | Jiuyuan | 44018.12 | 22912 | 455279386 | 10343 |
| Muchuan | Emei | 35880.64 | 19641 | 378253757 | 10542 |
| Muchuan | Shannan | 37688.95 | 20631 | 449289939 | 11921 |
| Muchuan | Diqing | 36980.92 | 21079 | 529825667 | 14327 |
| Muchuan | Pengxian | 42657.74 | 23549 | 638202409 | 14961 |
| Muchuan | Tianfu | 43069.75 | 23884 | 1170463489 | 27176 |
| Miyi | RJF | 40307.63 | 21913.5 | 58929748 | 1462 |
| Miyi | Shimian | 30211.98 | 17311 | 54834735 | 1815 |
| Miyi | Aba | 35163.92 | 18945 | 90441595 | 2572 |
| Miyi | Linzhi | 35939.77 | 18745.5 | 111628918 | 3106 |
| Miyi | Jiuyuan | 46161.44 | 25054.5 | 149286089 | 3234 |
| Miyi | Haiyan | 35738.44 | 20070 | 133089969 | 3724 |
| Miyi | Pengxian | 50645.67 | 25454 | 195239060 | 3855 |
| Miyi | Muchuan | 43005.05 | 23062 | 188921187 | 4393 |
| Miyi | Emei | 40505.64 | 20775 | 180695639 | 4461 |
| Miyi | Game fowl | 40846.30 | 21180.5 | 193774824 | 4744 |
| Miyi | Shannan | 41176.86 | 22600 | 212760830 | 5167 |
| Miyi | Tianfu | 45207.83 | 24656 | 236934238 | 5241 |
| Miyi | Diqing | 43298.98 | 22615 | 267024794 | 6167 |
| Miyi | Jinyang | 46214.89 | 23644 | 551574706 | 11935 |
| Miyi | Ganzi | 46204.40 | 26548.5 | 1548124626 | 33506 |
| Pengxian | RJF | 45312.35 | 23563 | 136344848 | 3009 |
| Pengxian | Miyi | 50645.67 | 25454 | 195239060 | 3855 |
| Pengxian | Haiyan | 48047.95 | 23601.5 | 278197639 | 5790 |
| Pengxian | Aba | 41608.69 | 23729 | 260345591 | 6257 |
| Pengxian | Linzhi | 37897.76 | 19863 | 288743050 | 7619 |
| Pengxian | Ganzi | 46984.29 | 24524 | 366383519 | 7798 |
| Pengxian | Shimian | 35634.92 | 19035.5 | 297337796 | 8344 |
| Pengxian | Jinyang | 48152.45 | 25457.5 | 425186137 | 8830 |
| Pengxian | Game fowl | 46737.79 | 23300 | 462610621 | 9898 |
| Pengxian | Diqing | 43601.38 | 23116 | 476737477 | 10934 |
| Pengxian | Emei | 43331.20 | 22180 | 554856030 | 12805 |
| Pengxian | Jiuyuan | 53219.51 | 26109 | 720379318 | 13536 |
| Pengxian | Shannan | 43205.33 | 23063 | 617360923 | 14289 |
| Pengxian | Muchuan | 42657.74 | 23549 | 638202409 | 14961 |
| Pengxian | Tianfu | 47731.83 | 25958 | 811011591 | 16991 |
| Shimian | RJF | 31885.59 | 16756 | 44129655 | 1384 |
| Shimian | Miyi | 30211.98 | 17311 | 54834735 | 1815 |
| Shimian | Haiyan | 29495.40 | 16828 | 100667805 | 3413 |
| Shimian | Ganzi | 32681.57 | 17448 | 111607548 | 3415 |
| Shimian | Aba | 30520.10 | 17387.5 | 110055495 | 3606 |
| Shimian | Jinyang | 32868.01 | 17920.5 | 122794875 | 3736 |
| Shimian | Game fowl | 31718.40 | 16786.5 | 140195343 | 4420 |
| Shimian | Linzhi | 26435.09 | 15864 | 142088625 | 5375 |
| Shimian | Jiuyuan | 32847.29 | 18059 | 184503208 | 5617 |
| Shimian | Diqing | 29186.64 | 17143.5 | 178038520 | 6100 |
| Shimian | Emei | 28477.80 | 16336 | 188095863 | 6605 |
| Shimian | Muchuan | 30800.21 | 17902 | 224625934 | 7293 |
| Shimian | Tianfu | 32435.78 | 18709 | 244857729 | 7549 |
| Shimian | Pengxian | 35634.92 | 19035.5 | 297337796 | 8344 |
| Shimian | Shannan | 27927.73 | 16692 | 251600931 | 9009 |
| Tianfu | RJF | 42851.85 | 22015 | 136911656 | 3195 |
| Tianfu | Miyi | 45207.83 | 24656 | 236934238 | 5241 |
| Tianfu | Haiyan | 38222.00 | 22021 | 230860888 | 6040 |
| Tianfu | Shimian | 32435.78 | 18709 | 244857729 | 7549 |
| Tianfu | Aba | 41585.59 | 23197 | 315717764 | 7592 |
| Tianfu | Linzhi | 34758.41 | 19421 | 267709296 | 7702 |
| Tianfu | Ganzi | 45818.70 | 24849 | 424785128 | 9271 |
| Tianfu | Game fowl | 43680.63 | 22628 | 432263515 | 9896 |
| Tianfu | Jinyang | 44435.16 | 23204 | 442574181 | 9960 |
| Tianfu | Emei | 40164.85 | 21805 | 479608459 | 11941 |
| Tianfu | Jiuyuan | 46099.26 | 25113 | 559137963 | 12129 |
| Tianfu | Shannan | 41712.96 | 22150 | 563124971 | 13500 |
| Tianfu | Pengxian | 47731.83 | 25958 | 811011591 | 16991 |
| Tianfu | Diqing | 38850.83 | 22443.5 | 669089042 | 17222 |
| Tianfu | Muchuan | 43069.75 | 23884 | 1170463489 | 27176 |
| Aba | RJF | 38063.38 | 19936.5 | 86708390 | 2278 |
| Aba | Miyi | 35163.92 | 18945 | 90441595 | 2572 |
| Aba | Shimian | 30520.10 | 17387.5 | 110055495 | 3606 |
| Aba | Emei | 33514.10 | 18361 | 155002721 | 4625 |
| Aba | Jiuyuan | 40012.01 | 22055 | 187736358 | 4692 |
| Aba | Jinyang | 36973.24 | 20188.5 | 189968502 | 5138 |
| Aba | Game fowl | 38261.70 | 20529 | 211242822 | 5521 |
| Aba | Pengxian | 41608.69 | 23729 | 260345591 | 6257 |
| Aba | Muchuan | 36978.20 | 20867.5 | 249380972 | 6744 |
| Aba | Tianfu | 41585.59 | 23197 | 315717764 | 7592 |
| Aba | Ganzi | 31420.56 | 17900 | 265095258 | 8437 |
| Aba | Diqing | 33482.48 | 19194.5 | 365092931 | 10904 |
| Aba | Linzhi | 27458.20 | 16669 | 326038615 | 11874 |
| Aba | Haiyan | 32149.70 | 18487 | 562105334 | 17484 |
| Aba | Shannan | 33991.95 | 19410.5 | 676711729 | 19908 |
| Diqing | RJF | 41014.49 | 20905 | 127432020 | 3107 |
| Diqing | Shimian | 29186.64 | 17143.5 | 178038520 | 6100 |
| Diqing | Miyi | 43298.98 | 22615 | 267024794 | 6167 |
| Diqing | Jiuyuan | 39038.52 | 21276 | 327103720 | 8379 |
| Diqing | Emei | 35019.45 | 19221 | 314369585 | 8977 |
| Diqing | Game fowl | 38818.27 | 21012 | 370830961 | 9553 |
| Diqing | Linzhi | 30900.14 | 17913 | 316201167 | 10233 |
| Diqing | Aba | 33482.48 | 19194.5 | 365092931 | 10904 |
| Diqing | Pengxian | 43601.38 | 23116 | 476737477 | 10934 |
| Diqing | Jinyang | 42288.50 | 21851.5 | 499088864 | 11802 |
| Diqing | Haiyan | 36324.05 | 20542 | 462913703 | 12744 |
| Diqing | Muchuan | 36980.92 | 21079 | 529825667 | 14327 |
| Diqing | Shannan | 37375.84 | 20783.5 | 632548666 | 16924 |
| Diqing | Tianfu | 38850.83 | 22443.5 | 669089042 | 17222 |
| Diqing | Ganzi | 41914.25 | 23045 | 856224240 | 20428 |
| Ganzi | RJF | 44531.37 | 21808 | 123975326 | 2784 |
| Ganzi | Shimian | 32681.57 | 17448 | 111607548 | 3415 |
| Ganzi | Linzhi | 33211.56 | 18618 | 192925972 | 5809 |
| Ganzi | Jiuyuan | 46223.15 | 23446.5 | 277893560 | 6012 |
| Ganzi | Emei | 39251.82 | 20228 | 295605431 | 7531 |
| Ganzi | Game fowl | 40727.84 | 21548 | 307902497 | 7560 |
| Ganzi | Pengxian | 46984.29 | 24524 | 366383519 | 7798 |
| Ganzi | Muchuan | 41891.38 | 22190.5 | 327925735 | 7828 |
| Ganzi | Aba | 31420.56 | 17900 | 265095258 | 8437 |
| Ganzi | Tianfu | 45818.70 | 24849 | 424785128 | 9271 |
| Ganzi | Shannan | 39268.90 | 21249 | 397519068 | 10123 |
| Ganzi | Haiyan | 33013.86 | 19348.5 | 430302654 | 13034 |
| Ganzi | Jinyang | 43104.62 | 22892 | 877523911 | 20358 |
| Ganzi | Diqing | 41914.25 | 23045 | 856224240 | 20428 |
| Ganzi | Miyi | 46204.40 | 26548.5 | 1548124626 | 33506 |
| Linzhi | RJF | 34818.60 | 17825 | 62360111 | 1791 |
| Linzhi | Miyi | 35939.77 | 18745.5 | 111628918 | 3106 |
| Linzhi | Game fowl | 36950.52 | 17938 | 181020575 | 4899 |
| Linzhi | Shimian | 26435.09 | 15864 | 142088625 | 5375 |
| Linzhi | Jiuyuan | 37379.15 | 19198 | 206893591 | 5535 |
| Linzhi | Ganzi | 33211.56 | 18618 | 192925972 | 5809 |
| Linzhi | Jinyang | 36593.09 | 19568 | 233207742 | 6373 |
| Linzhi | Emei | 30166.15 | 17256 | 198794939 | 6590 |
| Linzhi | Muchuan | 31905.49 | 18357 | 235303023 | 7375 |
| Linzhi | Pengxian | 37897.76 | 19863 | 288743050 | 7619 |
| Linzhi | Tianfu | 34758.41 | 19421 | 267709296 | 7702 |
| Linzhi | Haiyan | 29613.28 | 17273 | 282214549 | 9530 |
| Linzhi | Diqing | 30900.14 | 17913 | 316201167 | 10233 |
| Linzhi | Aba | 27458.20 | 16669 | 326038615 | 11874 |
| Linzhi | Shannan | 32192.91 | 19012 | 574418032 | 17843 |
| Haiyan | RJF | 35373.54 | 20366.5 | 96216042 | 2720 |
| Haiyan | Shimian | 29495.40 | 16828 | 100667805 | 3413 |
| Haiyan | Miyi | 35738.44 | 20070 | 133089969 | 3724 |
| Haiyan | Jiuyuan | 45038.68 | 20891.5 | 180965429 | 4018 |
| Haiyan | Emei | 34409.73 | 19099 | 152848018 | 4442 |
| Haiyan | Muchuan | 37848.27 | 20409 | 194767221 | 5146 |
| Haiyan | Pengxian | 48047.95 | 23601.5 | 278197639 | 5790 |
| Haiyan | Tianfu | 38222.00 | 22021 | 230860888 | 6040 |
| Haiyan | Jinyang | 36281.10 | 20168 | 271455173 | 7482 |
| Haiyan | Game fowl | 39297.02 | 20297 | 298696669 | 7601 |
| Haiyan | Linzhi | 29613.28 | 17273 | 282214549 | 9530 |
| Haiyan | Diqing | 36324.05 | 20542 | 462913703 | 12744 |
| Haiyan | Ganzi | 33013.86 | 19348.5 | 430302654 | 13034 |
| Haiyan | Aba | 32149.70 | 18487 | 562105334 | 17484 |
| Haiyan | Shannan | 32184.24 | 18711 | 1290941984 | 40111 |
| Shannan | RJF | 40118.53 | 21172 | 191846830 | 4782 |
| Shannan | Miyi | 41176.86 | 22600 | 212760830 | 5167 |
| Shannan | Shimian | 27927.73 | 16692 | 251600931 | 9009 |
| Shannan | Jinyang | 41740.78 | 22231 | 410520565 | 9835 |
| Shannan | Ganzi | 39268.90 | 21249 | 397519068 | 10123 |
| Shannan | Jiuyuan | 41306.99 | 21750 | 439712930 | 10645 |
| Shannan | Emei | 35796.59 | 19602 | 381949585 | 10670 |
| Shannan | Muchuan | 37688.95 | 20631 | 449289939 | 11921 |
| Shannan | Tianfu | 41712.96 | 22150 | 563124971 | 13500 |
| Shannan | Pengxian | 43205.33 | 23063 | 617360923 | 14289 |
| Shannan | Game fowl | 39370.66 | 20720 | 569654045 | 14469 |
| Shannan | Diqing | 37375.84 | 20783.5 | 632548666 | 16924 |
| Shannan | Linzhi | 32192.91 | 19012 | 574418032 | 17843 |
| Shannan | Aba | 33991.95 | 19410.5 | 676711729 | 19908 |
| Shannan | Haiyan | 32184.24 | 18711 | 1290941984 | 40111 |
| Game fowl | Shimian | 31718.40 | 16786.5 | 140195343 | 4420 |
| Game fowl | Miyi | 40846.30 | 21180.5 | 193774824 | 4744 |
| Game fowl | Linzhi | 36950.52 | 17938 | 181020575 | 4899 |
| Game fowl | Aba | 38261.70 | 20529 | 211242822 | 5521 |
| Game fowl | Emei | 39336.43 | 19946 | 269257831 | 6845 |
| Game fowl | Ganzi | 40727.84 | 21548 | 307902497 | 7560 |
| Game fowl | Haiyan | 39297.02 | 20297 | 298696669 | 7601 |
| Game fowl | Jinyang | 43609.40 | 21956 | 335617964 | 7696 |
| Game fowl | Jiuyuan | 43435.56 | 22247 | 335626550 | 7727 |
| Game fowl | Muchuan | 40555.55 | 20911 | 337706083 | 8327 |
| Game fowl | Diqing | 38818.27 | 21012 | 370830961 | 9553 |
| Game fowl | Tianfu | 43680.63 | 22628 | 432263515 | 9896 |
| Game fowl | Pengxian | 46737.79 | 23300 | 462610621 | 9898 |
| Game fowl | RJF | 46720.26 | 23445.5 | 637357814 | 13642 |
| Game fowl | Shannan | 39370.66 | 20720 | 569654045 | 14469 |

**Figure S8** Scatter diagrams showing the SNP counts in windows with sizes of 10, 20, 40, 100 and 200 kb.

**Figure S9** A summary of the selective sweep analysis of domestic chicken breeds.

**Figure S10** Plots of the distributions of the log2(θπ ratio) and zFST values for comparisons between the red jungle fowl and domestic chicken breeds.

**Table S6** Functional gene categories enriched for genes under selection in the 9 domestic chicken breeds.

| Breed | Category | Term | Term description | Gene Number | *P* Value | *Gene Symbols* |
| --- | --- | --- | --- | --- | --- | --- |
| Emei black fowls | GO-BP | GO:0015674 | di-, tri-valent inorganic cation transport | 5 | 0.04079 | *TRPC6, SLC40A1, S6, RYR2, CACNB2* |
|  | GO-MF | GO:0046873 | metal ion transmembrane transporter activity | 9 | 0.013814 | *KCND2, TRPC6, SLC40A1, S6, SCN5A, KCNK2, RYR2, KCNA4, CACNB2* |
|  | GO-MF | GO:0005261 | cation channel activity | 8 | 0.018509 | *KCND2, TRPC6, S6, SCN5A, KCNK2, RYR2, KCNA4, CACNB2* |
|  | GO-MF | GO:0005216 | ion channel activity | 10 | 0.021616 | *KCND2, TRPC6, S6, SCN5A, KCNK2, RYR2, KCNA4, CACNB2, GABRG1, GABRA2* |
|  | GO-MF | GO:0022838 | substrate specific channel activity | 10 | 0.022204 | *KCND2, TRPC6, S6, SCN5A, KCNK2, RYR2, KCNA4, CACNB2, GABRG1, GABRA2* |
|  | GO-MF | GO:0015267 | channel activity | 10 | 0.022804 | *KCND2, TRPC6, S6, SCN5A, KCNK2, RYR2, KCNA4, CACNB2, GABRG1, GABRA2* |
|  | GO-MF | GO:0022803 | passive transmembrane transporter activity | 10 | 0.022804 | *KCND2, TRPC6, S6, SCN5A, KCNK2, RYR2, KCNA4, CACNB2, GABRG1, GABRA2* |
|  | GO-MF | GO:0016597 | amino acid binding | 3 | 0.024875 | *ENSGALG00000003025, TPH2, ENSGALG00000021092, FTCD* |
|  | GO-MF | GO:0005262 | calcium channel activity | 4 | 0.026873 | *TRPC6, S6, RYR2, CACNB2* |
|  | GO-MF | GO:0003729 | mRNA binding | 3 | 0.036865 | *PTBP2, PIWIL1, IGF2BP3* |
|  | GO-MF | GO:0022836 | gated channel activity | 8 | 0.041351 | *KCND2, S6, SCN5A, RYR2, KCNA4, CACNB2, GABRG1, GABRA2* |
|  | INTERPRO | IPR009088 | Transcription factor IIA, beta-barrel | 2 | 0.035669 | *GTF2A2, GTF2A1* |
|  | INTERPRO | IPR006018 | Caldesmon and lymphocyte specific protein | 2 | 0.035669 | *LSP1, CALD1* |
|  | KEGG | gga04514 | Cell adhesion molecules (CAMs) | 6 | 0.037022 | *ITGB8, ITGA8, CD28, ALCAM, CDH2, CTLA4* |
| Jiuyuan black fowl | GO-BP | GO:0019932 | second-messenger-mediated signaling | 5 | 0.016598 | *TSHR, ENSGALG00000014182, PI4KA, CHRM4, ENSGALG00000014182* |
|  | GO-BP | GO:0040007 | growth | 7 | 0.025495 | *GDF8, GDF5, SLC1A2, CCNB2, ARID5B, PSEN1, ENSGALG00000004293* |
|  | GO-BP | GO:0006874 | cellular calcium ion homeostasis | 5 | 0.025709 | *RYR2, C3AR1, TRPC1, PSEN1, ENSGALG00000014182* |
|  | GO-BP | GO:0055074 | calcium ion homeostasis | 5 | 0.028374 | *RYR2, C3AR1, TRPC1, PSEN1, ENSGALG00000014182* |
|  | GO-BP | GO:0006470 | protein amino acid dephosphorylation | 7 | 0.030922 | *PTPN3, PTPRN2, PTPRS, PTPN5, PTPN14, PPM1E, PTPRM* |
|  | GO-BP | GO:0006875 | cellular metal ion homeostasis | 5 | 0.034181 | *RYR2, C3AR1, TRPC1, PSEN1, ENSGALG00000014182* |
|  | GO-BP | GO:0007155 | cell adhesion | 16 | 0.03421 | *CTNND1, NLGN4, PSEN1, CDH1, HES5, COL6A2, CNTNAP5, CDH8, FAT2, NRP1, ALCAM, LSAMP, CDH10, CD44, cad19, CDH13* |
|  | GO-BP | GO:0022610 | biological adhesion | 16 | 0.03421 | *CTNND1, NLGN4, PSEN1, CDH1, HES5, COL6A2, CNTNAP5, CDH8, FAT2, NRP1, ALCAM, LSAMP, CDH10, CD44, cad19, CDH13* |
|  | GO-BP | GO:0032103 | positive regulation of response to external stimulus | 4 | 0.038423 | *ENSGALG00000014182, BECN1, NTRK3, CDH13* |
|  | GO-BP | GO:0055065 | metal ion homeostasis | 5 | 0.040629 | *RYR2, C3AR1, TRPC1, PSEN1, ENSGALG00000014182* |
|  | GO-BP | GO:0032101 | regulation of response to external stimulus | 5 | 0.044095 | *ENSGALG00000014182, BECN1, ENSGALG00000004293, NTRK3, CDH13* |
|  | GO-MF | GO:0019904 | protein domain specific binding | 7 | 0.032191 | *QKI, NDFIP2, SH3BP2, PSEN1, U2AF1, CHMP2B, SRSF1* |
|  | GO-MF | GO:0019838 | growth factor binding | 6 | 0.033659 | *ACVR2A, NOV, IL1RAPL1, FLT4, NTRK3, IGF2R* |
|  | GO-MF | GO:0005509 | calcium ion binding | 23 | 0.033839 | *ENSGALG00000016257, UTRN, RYR2, MICU3, CDH1, EFHB, PLCH2, CDH8, CBLB, FAT2, SPARC, MICU2, TBC1D9B, ENTPD2, CDH10, PLS1, LETM1, cad19, FSTL4, ENSGALG00000004293, ANXA13, CDH13, SLIT3* |
|  | GO-MF | GO:0045296 | cadherin binding | 3 | 0.036931 | *CTNND1, PSEN1, CDH13* |
|  | INTERPRO | IPR017452 | GPCR, rhodopsin-like superfamily | 11 | 0.008465 | *DRD2, TSHR, OPN5, ENSGALG00000014182, OPN4-1, C3AR1, CCK1R, CHRM4, PRLHR, MC1R, ENSGALG00000014182* |
|  | INTERPRO | IPR000276 | 7TM GPCR, rhodopsin-like | 11 | 0.008949 | *DRD2, TSHR, OPN5, ENSGALG00000014182, OPN4-1, C3AR1, CCK1R, CHRM4, PRLHR, MC1R, ENSGALG00000014182* |
|  | INTERPRO | IPR000233 | Cadherin cytoplasmic region | 4 | 0.010849 | *CDH10, CDH1, cad19, CDH8* |
|  | INTERPRO | IPR002957 | Keratin, type I | 3 | 0.010879 | *KRT14, KRT15, KRT19* |
|  | INTERPRO | IPR013098 | Immunoglobulin I-set | 6 | 0.017856 | *LSAMP, FGFRL1, PTPRS, ROBO1, FSTL4, PRTG* |
|  | INTERPRO | IPR003598 | Immunoglobulin subtype 2 | 6 | 0.033763 | *LSAMP, FGFRL1, PTPRS, ROBO1, FSTL4, PRTG* |
|  | KEGG | gga04080 | Neuroactive ligand-receptor interaction | 16 | 0.016159 | *DRD2, CRHR1, ENSGALG00000014182, C3AR1, GRM8, PRLHR, MC1R, GABBR2, ENSGALG00000014182, TSHR, GRM7, CCK1R, CHRM4, ENSGALG00000004293, GPR156, GABARB* |
| Jinyang silky fowl | GO-BP | GO:0030534 | adult behavior | 7 | 0.001281 | *TSHR, CHRNA7, APP, CHRNA4, BBS2, PPT1, c-met* |
|  | GO-BP | GO:0007610 | behavior | 12 | 0.001973 | *TSHR, CHRNA7, CRHR1, SOBP, BDNF, LHX8, APP, CHRNA4, BBS2, MC4R, PPT1, c-met* |
|  | GO-BP | GO:0019932 | second-messenger-mediated signaling | 6 | 0.002191 | *TSHR, PIK3CA, CD36, RCAN3, GNB1, ENSGALG00000014182* |
|  | GO-BP | GO:0006928 | cell motion | 12 | 0.004873 | *CHRNA7, BDNF, APP, SEMA3C, Dab1, FLT1, LAMC1, CDH2, SLIT3, LMX1B, VAV3, COL5A1* |
|  | GO-BP | GO:0050890 | cognition | 10 | 0.008833 | *CHRNA7, SOBP, BDNF, LHX8, APP, CHRNA4, CRYAA, BBS2, PPT1, GNB1* |
|  | GO-BP | GO:0007611 | learning or memory | 5 | 0.012133 | *CHRNA7, BDNF, LHX8, APP, PPT1* |
|  | GO-BP | GO:0008283 | cell proliferation | 8 | 0.015927 | *HGF/SF, IFNAR2, TYR, RIPK2, LIPA, c-met, LMX1B, GNB1* |
|  | GO-BP | GO:0044093 | positive regulation of molecular function | 10 | 0.017631 | *TSHR, CHRNA7, HGF/SF, RIPK2, Dab1, c-met, GNB1, VAV3, EGF, ENSGALG00000014182* |
|  | GO-BP | GO:0046649 | lymphocyte activation | 7 | 0.018588 | *TSHR, CHRNA7, RIPK2, IFNAR1, BLNK, CBFB, DDOST* |
|  | GO-BP | GO:0032729 | positive regulation of interferon-gamma production | 3 | 0.019925 | *RIPK2, IFNAR1, SASH3* |
|  | GO-BP | GO:0001775 | cell activation | 8 | 0.020292 | *TSHR, CHRNA7, RIPK2, ENTPD2, IFNAR1, BLNK, CBFB, DDOST* |
|  | GO-BP | GO:0051674 | localization of cell | 9 | 0.020709 | *CHRNA7, SEMA3C, Dab1, FLT1, LAMC1, CDH2, LMX1B, VAV3, COL5A1* |
|  | GO-BP | GO:0048870 | cell motility | 9 | 0.020709 | *CHRNA7, SEMA3C, Dab1, FLT1, LAMC1, CDH2, LMX1B, VAV3, COL5A1* |
|  | GO-BP | GO:0043085 | positive regulation of catalytic activity | 9 | 0.022923 | *TSHR, CHRNA7, HGF/SF, Dab1, c-met, GNB1, VAV3, EGF, ENSGALG00000014182* |
|  | GO-BP | GO:0010517 | regulation of phospholipase activity | 3 | 0.026002 | *PPT1, GNB1, ENSGALG00000014182* |
|  | GO-BP | GO:0006461 | protein complex assembly | 8 | 0.031398 | *GTF2A2, GTF2A1, ENSGALG00000008404, CRYAA, ACTN2, GTF2B, LAMC1, VMA21* |
|  | GO-BP | GO:0070271 | protein complex biogenesis | 8 | 0.031398 | *GTF2A2, GTF2A1, ENSGALG00000008404, CRYAA, ACTN2, GTF2B, LAMC1, VMA21* |
|  | GO-BP | GO:0045321 | leukocyte activation | 7 | 0.032844 | *TSHR, CHRNA7, RIPK2, IFNAR1, BLNK, CBFB, DDOST* |
|  | GO-BP | GO:0043406 | positive regulation of MAP kinase activity | 4 | 0.033279 | *CHRNA7, HGF/SF, c-met, EGF* |
|  | GO-BP | GO:0001666 | response to hypoxia | 4 | 0.033279 | *CHRNA7, SLC2A8, MMP2, CHRNA4* |
|  | GO-BP | GO:0070482 | response to oxygen levels | 4 | 0.037378 | *CHRNA7, SLC2A8, MMP2, CHRNA4* |
|  | GO-BP | GO:0007267 | cell-cell signaling | 8 | 0.038241 | *CHRNA7, HGF/SF, BDNF, APP, GABRG1, CHRNA4, MC4R, PPT1* |
|  | GO-BP | GO:0060191 | regulation of lipase activity | 3 | 0.040042 | *PPT1, GNB1, ENSGALG00000014182* |
|  | GO-BP | GO:0032680 | regulation of tumor necrosis factor production | 3 | 0.040042 | *CHRNA7, RIPK2, SASH3* |
|  | GO-BP | GO:0050877 | neurological system process | 11 | 0.040996 | *CHRNA7, SOBP, BDNF, LHX8, APP, GABRG1, CHRNA4, CRYAA, BBS2, PPT1, GNB1* |
|  | GO-BP | GO:0000902 | cell morphogenesis | 8 | 0.042005 | *HGF/SF, BDNF, APP, LIPA, CRYAA, BBS2, LAMC1, SLIT3* |
|  | GO-BP | GO:0016477 | cell migration | 8 | 0.043973 | *SEMA3C, Dab1, FLT1, LAMC1, CDH2, LMX1B, VAV3, COL5A1* |
|  | GO-BP | GO:0033674 | positive regulation of kinase activity | 6 | 0.047716 | *CHRNA7, HGF/SF, Dab1, c-met, VAV3, EGF* |
|  | GO-MF | GO:0004896 | cytokine receptor activity | 5 | 0.013838 | *IFNAR2, LPR, IL1RAPL1, IFNAR1, IL10R2* |
|  | GO-MF | GO:0043394 | proteoglycan binding | 3 | 0.021564 | *GPC5, GPC6, COL5A1* |
|  | GO-MF | GO:0004623 | phospholipase A2 activity | 3 | 0.039971 | *ENSGALG00000016257, PLA2G4A, PLA2G10* |
|  | GO-MF | GO:0004222 | metalloendopeptidase activity | 6 | 0.049571 | *THSD4, MME, MMP16, MMP11, MMP2, MMP11* |
|  | INTERPRO | IPR008957 | Fibronectin, type III-like fold | 11 | 9.65E-05 | *IFNAR2, LPR, PTPRS, NEO1, EPHA3, EPHA5, IFNAR1, COL20A1, COL12A1, TYRO3, IL10R2* |
|  | INTERPRO | IPR003961 | Fibronectin, type III | 10 | 0.000361 | *LPR, PTPRS, NEO1, EPHA3, EPHA5, IFNAR1, COL20A1, COL12A1, TYRO3, IL10R2* |
|  | INTERPRO | IPR001818 | Peptidase M10A and M12B, matrixin and adamalysin | 3 | 0.012784 | *MMP16, MMP11, MMP2* |
|  | INTERPRO | IPR003129 | Laminin G, thrombospondin-type, N-terminal | 3 | 0.017542 | *COL20A1, COL12A1, COL5A1* |
|  | INTERPRO | IPR006026 | Peptidase, metallopeptidases | 3 | 0.017542 | *MMP16, MMP11, MMP2* |
|  | INTERPRO | IPR003962 | Fibronectin, type III subdomain | 4 | 0.018813 | *PTPRS, NEO1, EPHA3, EPHA5* |
|  | INTERPRO | IPR006029 | Neurotransmitter-gated ion-channel transmembrane region | 4 | 0.03519 | *CHRNA7, GABRQ, GABRG1, CHRNA4* |
|  | INTERPRO | IPR018000 | Neurotransmitter-gated ion-channel, conserved site | 4 | 0.039121 | *CHRNA7, GABRQ, GABRG1, CHRNA4* |
|  | INTERPRO | IPR006202 | Neurotransmitter-gated ion-channel ligand-binding | 4 | 0.039121 | *CHRNA7, GABRQ, GABRG1, CHRNA4* |
|  | INTERPRO | IPR006201 | Neurotransmitter-gated ion-channel | 4 | 0.043264 | *CHRNA7, GABRQ, GABRG1, CHRNA4* |
|  | KEGG | gga04080 | Neuroactive ligand-receptor interaction | 14 | 0.044081 | *CRHR1, ENSGALG00000020331, GRM8, LPAR4, CNR2, MC4R, GABBR2, ENSGALG00000014182, TSHR, F2RL3, LPR, GABRG1, TACR2, PARD3* |
|  | KEGG | gga04060 | Cytokine-cytokine receptor interaction | 10 | 0.048628 | *IFNLR1, HGF/SF, IFNAR2, LPR, IFNAR1, FLT1, IL22RA1, c-met, IL10R2, EGF* |
| Muchuan black-bone fowl | GO-BP | GO:0051336 | regulation of hydrolase activity | 7 | 0.021843 | *RABGAP1L, FOXL2, IAP3, TBC1D1, TBC1D22B, AGFG1, ENSGALG00000014182* |
|  | GO-BP | GO:0006470 | protein amino acid dephosphorylation | 6 | 0.023641 | *DUPD1, ENSGALG00000020775, ENSGALG00000023842, PTPN5, PTPRQ, PTPRO* |
|  | GO-BP | GO:0016311 | dephosphorylation | 6 | 0.043098 | *DUPD1, ENSGALG00000020775, ENSGALG00000023842, PTPN5, PTPRQ, PTPRO* |
|  | GO-MF | GO:0004721 | phosphoprotein phosphatase activity | 8 | 0.004043 | *DUPD1, ENSGALG00000020775, ENSGALG00000023842, PTPN5, CTDSPL2, PTPRQ, PTPRO, CTDSPL* |
|  | GO-MF | GO:0016791 | phosphatase activity | 9 | 0.013464 | *DUPD1, ENSGALG00000020775, ENSGALG00000023842, PTPN5, CTDSPL2, ACP6, PTPRQ, PTPRO, CTDSPL* |
|  | GO-MF | GO:0004725 | protein tyrosine phosphatase activity | 6 | 0.015857 | *DUPD1, ENSGALG00000020775, ENSGALG00000023842, PTPN5, PTPRQ, PTPRO* |
|  | GO-MF | GO:0004674 | protein serine/threonine kinase activity | 12 | 0.020907 | *BRSK2, MAST2, STK16, TLK2, MAPK6, SRPK1, AKT3, CDK14, TBC1D22B, RPS6KA5, MAPK14, SBK2* |
|  | INTERPRO | IPR011948 | Dullard-like phosphatase domain | 2 | 0.047271 | *CTDSPL2, CTDSPL* |
| Miyi fowl | GO-BP | GO:0018195 | peptidyl-arginine modification | 4 | 0.000193 | *PADI2, PADI3, PRMT7, PADI1* |
|  | GO-BP | GO:0000052 | citrulline metabolic process | 3 | 0.003969 | *PADI2, PADI3, PADI1* |
|  | GO-BP | GO:0018101 | peptidyl-citrulline biosynthetic process from peptidyl-arginine | 3 | 0.003969 | *PADI2, PADI3, PADI1* |
|  | GO-BP | GO:0019240 | citrulline biosynthetic process | 3 | 0.003969 | *PADI2, PADI3, PADI1* |
|  | GO-BP | GO:0040029 | regulation of gene expression, epigenetic | 5 | 0.016253 | *H2AFY, PRMT7, SIRT3, DICER1, ATF7IP* |
|  | GO-BP | GO:0030035 | microspike assembly | 3 | 0.025191 | *CDC42, ACTN2, MTSS1* |
|  | GO-BP | GO:0006396 | RNA processing | 11 | 0.032138 | *GEMIN2, ADARB2, LARP7, DROSHA, ZFC3H1, INTS9, PRMT7, YBX1, EXOSC9, DICER1, PRPF6* |
|  | GO-BP | GO:0016071 | mRNA metabolic process | 7 | 0.034012 | *GEMIN2, DROSHA, PRMT7, YBX1, PNRC2, EIF3E, PRPF6* |
|  | GO-MF | GO:0016813 | hydrolase activity, acting on carbon-nitrogen (but not peptide) bonds, in linear amidines | 4 | 0.001485 | *PADI2, PADI3, PADI1, DDAH1* |
|  | GO-MF | GO:0004668 | protein-arginine deiminase activity | 3 | 0.003844 | *PADI2, PADI3, PADI1* |
|  | GO-MF | GO:0005509 | calcium ion binding | 26 | 0.00933 | *CDH23, PPP2R3C, LRP1B, CDH5, ACTN2, CDHR5, RHBDL3, CLSTN2, CANX, SMOC2, TNNC1, kcnma1, UTRN, PCDH18, ANXA5, OIT3, CDH2, EDEM3, DMD, PKD2L2, PLA2G4A, PADI2, PADI3, RELN, PADI1, CRELD2* |
|  | GO-MF | GO:0043169 | cation binding | 77 | 0.034208 | *CDH23, DEAF1, USP16, BAZ1A, ZDHHC13, CSRP3, ALPL, CDH5, ESRRB, ACTN2, PARK2, KDM4A, RHBDL3, CANX, TNNC1, CYB5A, RUFY1, GBE1, UTRN, CH25H, ADGB, ADAMTS2, LTN1, ANXA5, OIT3, CDH2, ZNF827, PLA2G4A, ESR1, PADI2, ZZZ3, SIRT3, SLC30A7, RNF220, GTF2B, NOX3, PADI1, CRELD2, GALC, MOCS1, CTBS, PPP2R3C, ENSGALG00000006013, LRP1B, RNF139, FUCA1, SMYD3, CDHR5, ZGRF1, ENSGALG00000014148, AGL, CLSTN2, ZNF410, LMO4, Fe, SMOC2, SH3RF1, kcnma1, GATA5, FBXO30, PCDH18, [4Fe-4S], TRAF6, DCT, AMY1A, ZDHHC6, HMGCL, EDEM3, DMD, PKD2L2, PHF21A, CYP2H1, PADI3, ZNF319, RELN, DICER1, BCL11A* |
|  | GO-MF | GO:0043167 | ion binding | 77 | 0.037253 | *CDH23, DEAF1, USP16, BAZ1A, ZDHHC13, CSRP3, ALPL, CDH5, ESRRB, ACTN2, PARK2, KDM4A, RHBDL3, CANX, TNNC1, CYB5A, RUFY1, GBE1, UTRN, CH25H, ADGB, ADAMTS2, LTN1, ANXA5, OIT3, CDH2, ZNF827, PLA2G4A, ESR1, PADI2, ZZZ3, SIRT3, SLC30A7, RNF220, GTF2B, NOX3, PADI1, CRELD2, GALC, MOCS1, CTBS, PPP2R3C, ENSGALG00000006013, LRP1B, RNF139, FUCA1, SMYD3, CDHR5, ZGRF1, ENSGALG00000014148, AGL, CLSTN2, ZNF410, LMO4, Fe, SMOC2, SH3RF1, kcnma1, GATA5, FBXO30, PCDH18, [4Fe-4S], TRAF6, DCT, AMY1A, ZDHHC6, HMGCL, EDEM3, DMD, PKD2L2, PHF21A, CYP2H1, PADI3, ZNF319, RELN, DICER1, BCL11A* |
|  | INTERPRO | IPR017452 | GPCR, rhodopsin-like superfamily | 11 | 0.011367 | *TSHR, LPAR3, DRD4, PTGFR, VT4, SS1R, CCR6, NPY6R, MC4R, GPR103, TRHR* |
|  | INTERPRO | IPR000276 | 7TM GPCR, rhodopsin-like | 11 | 0.012001 | *TSHR, LPAR3, DRD4, PTGFR, VT4, SS1R, CCR6, NPY6R, MC4R, GPR103, TRHR* |
|  | INTERPRO | IPR001846 | von Willebrand factor, type D | 3 | 0.030897 | *VTG2, VTG1, ENSGALG00000006717* |
|  | KEGG | gga04080 | Neuroactive ligand-receptor interaction | 18 | 0.005015 | *LPAR3, ADRA1B, DRD4, GABRA4, PTGFR, GluR1/A, NMUR2, CNR2, MC4R, TRHR, TSHR, VT4, LPR, GABRG3, SS1R, GRIA4, NPY6R, GRM1* |
| Pengxian yellow fowl | GO-BP | GO:0007267 | cell-cell signaling | 9 | 0.015446 | *DMD, C1H2ORF49, BDNF, ENSGALG00000015966, GABRG2, APP, IL6, FZD1, LIN7B* |
|  | GO-BP | GO:0006928 | cell motion | 11 | 0.016054 | *BDNF, PLAU, chAnk3, APP, SEMA3C, Dab1, kif5c, LRP8, SLIT3, CDH13, VAV3* |
|  | GO-BP | GO:0007610 | behavior | 10 | 0.020544 | *TSHR, SOBP, BDNF, GABRG2, APP, LSP1, ZIC1, ESPN, ZIC4, PBX3* |
|  | GO-BP | GO:0045785 | positive regulation of cell adhesion | 4 | 0.020774 | *CD47, cdk6, CDH13, VAV3* |
|  | GO-BP | GO:0007411 | axon guidance | 5 | 0.04013 | *BDNF, chAnk3, APP, kif5c, SLIT3* |
|  | GO-BP | GO:0000904 | cell morphogenesis involved in differentiation | 7 | 0.040957 | *BDNF, ENSGALG00000015966, chAnk3, APP, kif5c, CRYAA, SLIT3* |
|  | GO-BP | GO:0000725 | recombinational repair | 3 | 0.042 | *RAD52, SHFM1, BRCA1* |
|  | GO-BP | GO:0046716 | muscle maintenance | 3 | 0.042 | *DMD, LARGE, IL6* |
|  | GO-BP | GO:0000724 | double-strand break repair via homologous recombination | 3 | 0.042 | *RAD52, SHFM1, BRCA1* |
|  | GO-BP | GO:0007409 | axonogenesis | 6 | 0.043633 | *BDNF, ENSGALG00000015966, chAnk3, APP, kif5c, SLIT3* |
|  | GO-BP | GO:0016192 | vesicle-mediated transport | 10 | 0.04607 | *APP, ITGAV, TSNARE1, RER1, LRP8, SCFD1, VAV3, LIN7B, CHMP7, AKTIP* |
|  | GO-BP | GO:0050877 | neurological system process | 11 | 0.047557 | *DMD, SOBP, OPN5, BDNF, ENSGALG00000015966, GABRG2, APP, MBP, CRYAA, PBX3, LIN7B* |
|  | GO-BP | GO:0048812 | neuron projection morphogenesis | 6 | 0.049334 | *BDNF, ENSGALG00000015966, chAnk3, APP, kif5c, SLIT3* |
|  | GO-BP | GO:0019226 | transmission of nerve impulse | 6 | 0.049334 | *DMD, ENSGALG00000015966, GABRG2, APP, MBP, LIN7B* |
|  | GO-MF | GO:0046872 | metal ion binding | 69 | 0.033785 | *ENSGALG00000016257, TRIM37, BRCA1, ITGAV, EFCAB1, ELMSAN1, ENSGALG00000009006, MBNL1, ABLIM1, ZNF276, TBXAS1, UBR1, U2AF1, ATP11A, CYB5A, MAP3K7, NR6A1, HIVEP2, ADGRL4, HCCS, EYA4, SOBP, ZZZ3, ENTPD2, NECAB3, ZIC1, ZIC4, COQ7, MAN1A2, LRP8, NOX3, CRELD2, CAPN3, MOCS1, LRP1B, PRMT3, RUFY2, ANKIB1, F2, PLCH2, SMYD3, MCFD2, SPOCK1, KCNJ3, ENSGALG00000014148, CBFA2T2, ZC3H15, ZBTB48, SLIT3, CDH13, PTGR2, VAV3, FHL2, RYR2, BBOX1, ENSGALG00000006608, CYP19A1, VAT1, SGCE, ZNF804A, DMD, NHLRC1, ZFAT, HAAO, PHF21A, KDM1B, MYCBP2, NR5A1, PLOD2* |
|  | GO-MF | GO:0043167 | ion binding | 70 | 0.034612 | *ENSGALG00000016257, TRIM37, BRCA1, ITGAV, EFCAB1, ELMSAN1, ENSGALG00000009006, MBNL1, ABLIM1, ZNF276, TBXAS1, UBR1, U2AF1, ATP11A, CYB5A, MAP3K7, NR6A1, HIVEP2, ADGRL4, HCCS, EYA4, SOBP, ZZZ3, ENTPD2, NECAB3, ZIC1, ZIC4, COQ7, MAN1A2, LRP8, NOX3, CRELD2, CAPN3, MOCS1, LRP1B, GABRG2, PRMT3, ANKIB1, RUFY2, F2, PLCH2, SMYD3, MCFD2, SPOCK1, KCNJ3, ENSGALG00000014148, CBFA2T2, ZC3H15, ZBTB48, SLIT3, CDH13, PTGR2, VAV3, FHL2, RYR2, BBOX1, ENSGALG00000006608, CYP19A1, VAT1, SGCE, ZNF804A, DMD, NHLRC1, ZFAT, HAAO, PHF21A, KDM1B, MYCBP2, NR5A1, PLOD2* |
|  | GO-MF | GO:0043169 | cation binding | 69 | 0.044391 | *ENSGALG00000016257, TRIM37, BRCA1, ITGAV, EFCAB1, ELMSAN1, ENSGALG00000009006, MBNL1, ABLIM1, ZNF276, TBXAS1, UBR1, U2AF1, ATP11A, CYB5A, MAP3K7, NR6A1, HIVEP2, ADGRL4, HCCS, EYA4, SOBP, ZZZ3, ENTPD2, NECAB3, ZIC1, ZIC4, COQ7, MAN1A2, LRP8, NOX3, CRELD2, CAPN3, MOCS1, LRP1B, PRMT3, RUFY2, ANKIB1, F2, PLCH2, SMYD3, MCFD2, SPOCK1, KCNJ3, ENSGALG00000014148, CBFA2T2, ZC3H15, ZBTB48, SLIT3, CDH13, PTGR2, VAV3, FHL2, RYR2, BBOX1, ENSGALG00000006608, CYP19A1, VAT1, SGCE, ZNF804A, DMD, NHLRC1, ZFAT, HAAO, PHF21A, KDM1B, MYCBP2, NR5A1, PLOD2* |
|  | KEGG | gga04310 | Wnt signaling pathway | 10 | 0.038909 | *WNT9a, NFATC3, TCF1, GSK3B, DAAM2, CSNK2A1, MAP3K7, FZD1, SKP1, MYC* |
| Shimian caoke fowl | GO-BP | GO:0001822 | kidney development | 5 | 0.015483 | *SGPL1, SALL1, GLI3, SLIT2, ENSGALG00000014182* |
|  | GO-BP | GO:0001655 | urogenital system development | 5 | 0.022594 | *SGPL1, SALL1, GLI3, SLIT2, ENSGALG00000014182* |
|  | GO-MF | GO:0008081 | phosphoric diester hydrolase activity | 5 | 0.01129 | *PDE5A, PDE7A, PLCXD1, PDE10A, PLCL2* |
|  | GO-MF | GO:0004114 | 3',5'-cyclic-nucleotide phosphodiesterase activity | 3 | 0.048584 | *PDE5A, PDE7A, PDE10A* |
|  | KEGG | gga04514 | Cell adhesion molecules (CAMs) | 8 | 0.009646 | *ITGB8, CD226, CD28, VCAM1, NEO1, CDH4, CTLA4, NRXN3* |
|  | KEGG | gga04080 | Neuroactive ligand-receptor interaction | 13 | 0.010926 | *GRIN2B, GLP-1R, FSH, SS5R, GABRG3, PRLHR, F2, GRM8, MC4R, TAAR5, ENSGALG00000006642, GABARB, ENSGALG00000014182* |
|  | KEGG | gga00770 | Pantothenate and CoA biosynthesis | 3 | 0.027297 | *UPB1, PANK1, DPYD* |
|  | KEGG | gga00230 | Purine metabolism | 8 | 0.03404 | *ADCY1, PDE5A, PDE10A, PDE9A, NT5C3A, AK5, PPAT, PAICS* |
| Tianfu black-bone fowl | GO-BP | GO:0007017 | microtubule-based process | 8 | 0.017764 | *TUBG1, BRCA1, DNAH5, KIF18A, CRYAA, TUBB3, NEK2, ENSGALG00000000433* |
|  | GO-BP | GO:0051216 | cartilage development | 5 | 0.023328 | *BARX2B, TGFB2, SOX5, HIF1A, ENSGALG00000007509* |
|  | GO-BP | GO:0045639 | positive regulation of myeloid cell differentiation | 3 | 0.026282 | *KITLG, HIF1A, ENSGALG00000007509* |
|  | GO-BP | GO:0045597 | positive regulation of cell differentiation | 7 | 0.026354 | *PPAR, BDNF, KITLG, BMPR-II, TGFB2, HIF1A, ENSGALG00000007509* |
|  | GO-BP | GO:0006366 | transcription from RNA polymerase II promoter | 4 | 0.027651 | *GTF2A2, GTF2A1, MED17, BMPR-II* |
|  | GO-BP | GO:0048771 | tissue remodeling | 4 | 0.030945 | *ATG5, LIPA, TGFB2, HIF1A* |
|  | GO-BP | GO:0006367 | transcription initiation from RNA polymerase II promoter | 3 | 0.03866 | *GTF2A2, GTF2A1, MED17* |
|  | GO-BP | GO:0006112 | energy reserve metabolic process | 3 | 0.045525 | *AGL, PHKB, ENSGALG00000007509* |
|  | GO-MF | GO:0005506 | iron ion binding | 11 | 0.017393 | *FADS1, HAAO, CH25H, INOS, ENSGALG00000000641, BBOX1, CYB5B, FADS2, CYB5A, HCCS, PPP2CA* |
|  | GO-MF | GO:0030695 | GTPase regulator activity | 10 | 0.043543 | *RASA3, GRTP1, FBXO8, TBC1D5, PSD, TBC1D8B, SRGAP1, TRIO, NF1, DOCK2* |
|  | INTERPRO | IPR001199 | Cytochrome b5 | 3 | 0.010741 | *CYB5B, FADS2, CYB5A* |
|  | KEGG | gga04310 | Wnt signaling pathway | 11 | 0.009236 | *WNT8b, VANGL2, NFATC3, ROCK2, TCF1, TCF7L2, NFAT5, PRICKLE1, SKP1, MYC, PPP2CA* |
| Xishuangbanna game fowl | GO-BP | GO:0019438 | aromatic compound biosynthetic process | 4 | 0.001478 | *MOCS1, TPK1, PTS, GPHN* |
|  | GO-BP | GO:0042559 | pteridine and derivative biosynthetic process | 3 | 0.014163 | *MOCS1, PTS, GPHN* |
|  | GO-BP | GO:0044271 | nitrogen compound biosynthetic process | 12 | 0.019461 | *ADCY1, MOCS1, INOS, TPK1, PADI3, PTS, ATP13A5, GPHN, SNCA, PADI1, ATP13A4, ATP2B1* |
|  | GO-BP | GO:0042558 | pteridine and derivative metabolic process | 3 | 0.023426 | *MOCS1, PTS, GPHN* |
|  | GO-BP | GO:0055001 | muscle cell development | 4 | 0.023573 | *MYH11, APP, ATG5, GPHN* |
|  | GO-BP | GO:0008344 | adult locomotory behavior | 4 | 0.026415 | *TSHR, APP, PPT1, SNCA* |
|  | GO-BP | GO:0018130 | heterocycle biosynthetic process | 4 | 0.03952 | *MOCS1, TPK1, PTS, GPHN* |
|  | GO-BP | GO:0007017 | microtubule-based process | 7 | 0.040667 | *TUBG1, BRCA1, APP, KIF18A, KIF20A, KIF4A, KIF21B* |
|  | GO-BP | GO:0048747 | muscle fiber development | 3 | 0.040716 | *MYH11, APP, GPHN* |
|  | GO-MF | GO:0005516 | calmodulin binding | 6 | 0.000699 | *INOS, MYH11, CAMK2D, CALD1, ATP2B1, PHKB* |
|  | GO-MF | GO:0003779 | actin binding | 9 | 0.005703 | *SYNE1, ARPC1A, MYH11, ARPC1B, VCL, ACTN2, FHOD3, CALD1, AFAP1* |
|  | GO-MF | GO:0000166 | nucleotide binding | 49 | 0.006198 | *ACTG2, PRKCB, TUBG1, MAP3K5, MYH11, GPD1L, KIF20A, RAB33A, IGF2BP1, ESRP2, KIF21B, SRSF10, AIFM1, CHD9, DUS2, TPK1, ENSGALG00000002880, RAB24, EGFR, CAMK2D, NDUFA10, KIF18A, ATP13A5, VRK3, KIF4A, MAP3K7, ENOX2, HBS1L, GNAO1, DOCK4, STK17A, CPEB2, NLK, MARS2, MAPK14, ATP2B1, MAP3K4, ENSGALG00000007516, INOS, FASTKD3, SCAF8, MTRR, RND2, GPHN, DDX28, GRK6, ATP13A4, ERO1B, PI4K2B* |
|  | GO-MF | GO:0008092 | cytoskeletal protein binding | 10 | 0.025004 | *SYNE1, ARPC1A, MYH11, ARPC1B, VCL, ACTN2, SNCA, FHOD3, CALD1, AFAP1* |
|  | GO-MF | GO:0017076 | purine nucleotide binding | 39 | 0.04167 | *ACTG2, PRKCB, TUBG1, MAP3K5, MYH11, KIF20A, RAB33A, KIF21B, AIFM1, CHD9, DUS2, TPK1, ENSGALG00000002880, RAB24, EGFR, CAMK2D, NDUFA10, KIF18A, ATP13A5, VRK3, KIF4A, MAP3K7, HBS1L, GNAO1, DOCK4, STK17A, NLK, MARS2, MAPK14, ATP2B1, MAP3K4, INOS, FASTKD3, RND2, DDX28, GRK6, ATP13A4, ERO1B, PI4K2B* |
|  | INTERPRO | IPR008942 | ENTH/VHS | 3 | 0.027084 | *CLINT1, RPRD1A, GGA3* |
|  | INTERPRO | IPR017383 | Actin-related protein 2/3 complex, subunit 1 | 2 | 0.047271 | *ARPC1A, ARPC1B* |
|  | KEGG | gga04520 | Adherens junction | 7 | 0.011303 | *EGFR, EP300, VCL, TCF7L2, ACTN2, NLK, MAP3K7* |
|  | KEGG | gga04912 | GnRH signaling pathway | 7 | 0.022227 | *ADCY1, FSH, EGFR, CAMK2D, MAPK13, MAPK14, MAP3K4* |
|  | KEGG | gga04270 | Vascular smooth muscle contraction | 7 | 0.036804 | *ADCY1, ACTG2, PRKCB, RAMP2, KCNMB4, MBSP, CALD1* |
|  | KEGG | gga04010 | MAPK signaling pathway | 12 | 0.037947 | *FAS, PRKCB, BDNF, MAP3K5, FGF14, CACNA2D3, EGFR, MAPK13, NLK, MAP3K7, MAPK14, MAP3K4* |
|  | KEGG | gga04622 | RIG-I-like receptor signaling pathway | 5 | 0.042996 | *ATG5, MAPK13, TRADD, MAP3K7, MAPK14* |

Note: Only GO-BP, GO-MF, KEGG pathway or InterPro domain terms with a P value less than 0.05 are listed. P-values are corrected by the Benjamini-Hochberg FDR.

**Table S7** The general phenotypic differences between RJF, Tibetan chickens and local Sichuan chicken breeds.

| Breed | Red jungle fowl | Tibetan fowl | Miyi fowl | Emei black fowl | Muchuan black-bone fowl | Shimian caoke fowl | Tianfu black-bone fowl | Jinyang silky fowl | Pengxian yellow fowl | Jiuyuan black-bone fowl |
| --- | --- | --- | --- | --- | --- | --- | --- | --- | --- | --- |
| Place of origin | Yunnan | Qinghai-Tibet Plateau | Szechwan Basin | Szechwan Basin | Szechwan Basin | Szechwan Basin | Szechwan Basin | Szechwan Basin | Szechwan Basin | Szechwan Basin |
| Colour (♂) | Golden, red, black blue | Golden,red, black blue | Red, black | Black, reddish black | Black | Black, red | Black | White | Yellow, red, black | Black, reddish black |
| Skin colour (♂) | White | White | White | White | Corvinus | White | Corvinus | White | White | Corvinus |
| Birth weight (♂) (g) | 26 | 30 | — | 40 | — | 41 | 31 | — | 40 | — |
| Sexual maturity (♂) | 120 | 120 | 120 | — | 200 | 208 | — | 120 | 120 | — |
| Age at first egg (♀) (day) | 252.27 | 232.06 | 191.85 | 179.28 | 187.47 | 157.45 | 148.76 | 156.73 | 169.54 | 181.32 |
| Body weight at first egg (♀) (g) | 860.68 | 1120.45 | 1793.67 | 1896.52 | 2194.14 | 2215.77 | 1423.62 | 1195.84 | 1438.86 | 1582.29 |
| Egg number at 300 days of age (♀) | 22.52 | 42.75 | 57.29 | 83.34 | 78.25 | 98.93 | 102.46 | 67.57 | 91.69 | 73.63 |
| Egg weight at 300 days of age (♀) (g) | 29.15 | 34.97 | 54.35 | 53.81 | 54.03 | 55.43 | 55.43 | 52.47 | 54.52 | 55.62 |
| Body weight at 180 days of age (♂) (g) | 1113.4 | 1225.22 | 2178.06 | 2642.3 | 2484.3 | 3278.25 | 2485.67 | 2090 | 2032.35 | 2238.81 |
| Body weight at 180 days of age (♀) (g) | 780.65 | 1085.52 | 1517.65 | 1889.5 | 2031.86 | 2727.95 | 1594.51 | 1340.45 | 1524.28 | 1567.06 |
| Whole net carcass rate (♂) (%) | — | 73.28 | 77.7 | 79.65 | 78.9 | 69.5 | — | — | 79.1 | 79.7 |
| Whole net carcass rate (♀) (%) | — | 72.17 | 70.4 | 70.9 | 69.4 | 65.2 | — | — | 72.12 | 67 |
| Adult weight (♂) (g) | — | 1490 | 2405 | 2622 | 2680 | 3650 | — | 2230 | 3950 | 2615.5 |
| Adult weight (♀) (g) | — | 1150 | 1985 | 1904 | 2290 | 3060 | — | 1450 | 1880 | 1762.5 |
| Body slanting length (♂) (cm) | — | 21.2 | 25.62 | 26.3 | 28.2 | 25.4 | — | 24.9 | 24.4 | 28.8 |
| Body slanting length (♀) (cm) | — | 19.2 | 23 | 22.1 | 18.22 | 22.4 | — | 21.1 | 20.5 | 24.2 |
| Chest breadth (♂) (cm) | — | 7.2 | 9.19 | 8.5 | 9.08 | 9.6 | — | 8.6 | 8.1 | 9.4 |
| Chest breadth (♀) (cm) | — | 7.01 | 7.31 | 6.9 | 7.44 | 8.7 | — | 6.5 | 6.7 | 8 |
| Chest depth (♂) (cm) | — | 6.4 | 11.6 | 12.6 | 9.02 | 11.2 | — | 11.7 | 11.9 | 11.8 |
| Chest depth (♀) (cm) | — | 6 | 10.72 | 10.3 | 8.54 | 8.7 | — | 10.1 | 9.9 | 11 |
| Pelvis width (♂) (cm) | — | 7.6 | 8.46 | 9.6 | 9.08 | 11.3 | — | 8.7 | 9.9 | 9.6 |
| Pelvis width (♀) (cm) | — | 7.3 | 7.88 | 8.3 | 8.18 | 11.1 | — | 7.7 | 8.2 | 8.72 |
| Shank length (♂) (cm) | — | 9.5 | 12.21 | 13.9 | 11.5 | 14.8 | — | 12 | 12.3 | 13.1 |
| Shank length (♀) (cm) | — | 8.5 | 9.9 | 10.1 | 8.99 | 11.3 | — | 9.1 | 9.5 | 10.6 |

Note: Body slanting length, chest breadth, chest depth, pelvis width and shank length traits all refer to adults. All phenotypic data were collected from references or Sichuan local varieties. “—” indicates unavailable data.

**Table S8** The candidate genes associated with black bones and skin.

“-” indicates novel genes in chicken. **(a) The candidate genes associated with black-bone (skin) identified in the three black-bone chicken population genomes compared with RJFs.** Only genes shared in three black-bone chicken populations and did not present in other populations were listed.

| Chro. | Gene symbols | Gene Name | *zF_ST_* in Tianfu | *zFST* in Muchuan | *zFST* in Jiuyuan |
| --- | --- | --- | --- | --- | --- |
| 1 | *AEBP2* | AE binding protein 2 | 2.41 | 3.17 | 2.88 |
| 1 | *CHMP2B* | Chromatin modifying protein 2B | 3.20 | 2.94 | 2.86 |
| 1 | *PLEKHA5* | Pleckstrin homology domain containing, family A member 5 | 3.26 | 3.36 | 3.28 |
| 1 | *PIT-1* | POU domain, class 1, Transcription factor 1 (Pit1, growth hormone factor 1) | 3.20 | 2.94 | 2.86 |
| 1 | *-* | - | 3.48 | 3.19 | 3.22 |
| 2 | *6-Mar* | Membrane-associated ring finger (C3HC4) 6, E3 ubiquitin protein ligase | 2.28 | 3.45 | 2.21 |
| 2 | *LOC428499* | - | 2.28 | 3.23 | 2.21 |
| 2 | *-* | - | 2.18 | 3.18 | 2.35 |
| 5 | *RPS6KA5* | Ribosomal protein S6 kinase, 90kDa, polypeptide 5 | 5.22 | 4.75 | 4.33 |
| 11 | *DBNDD1* | Dysbindin (dystrobrevin binding protein 1) domain containing 1 | 4.16 | 4.46 | 4.58 |
| 11 | *MC1R* | melanocortin 1 receptor (alpha Melanocyte stimulating hormone receptor) | 4.16 | 4.46 | 4.58 |
| 11 | *SPIRE2* | Spire homolog 2 (Drosophila) | 3.35 | 3.13 | 3.09 |
| 11 | *TCF25* | Transcription factor 25 (basic helix-loop-helix) | 4.16 | 4.46 | 4.58 |
| 11 | *TUBB3* | Tubulin beta-4 chain | 4.16 | 4.46 | 4.58 |
| 11 | *DEF8* | differentially expressed in FDCP 8 homolog | 4.16 | 4.46 | 4.58 |
| 11 | *-* | - | 2.70 | 3.13 | 3.09 |
| 12 | *TAMM41* | Chromosome 3 open reading frame 31 | 2.38 | 2.09 | 2.25 |
| 20 | *UQCC* | Ubiquinol-cytochrome c reductase complex chaperone, CBP3 homolog (yeast) | 2.27 | 2.90 | 2.46 |
| 20 | *LOC100859111* | - | 2.27 | 2.90 | 2.46 |
| LGE64 | *LIG1* | Similar to ligase I | 3.60 | 2.31 | 2.06 |
| LGE64 | *ZNF628* | Zinc finger protein 628 | 2.42 | 2.31 | 2.06 |
| LGE64 | *-* | - | 3.60 | 2.31 |  |

**(b) The candidate genes associated with black-bone (skin) identified in the three black-bone chicken population genomes compared with othernon-black-bone Sichuan domestic chickens.** The top 50 genes with highest *zF_ST_* scores were listed.

| Gene  name | *zF_ST_* | Gene name | *zF_ST_* | Gene  name | *zF_ST_* | Gene  name | *zF_ST_* | Gene  name | *zF_ST_* |
| --- | --- | --- | --- | --- | --- | --- | --- | --- | --- |
| *ELMO2* | 8.26 | *SLMO2* | 6.27 | *CTSZ* | 5.33 | *ERCC8* | 5.02 | *INOS* | 4.20 |
| *SNRB'* | 8.26 | *SEMA3A* | 6.05 | *TH1L* | 5.33 | *ANKRD31* | 5.01 | *cad19* | 4.19 |
| *DDX27* | 8.26 | *SPIRE2* | 6.03 | *gga-mir-6597* | 5.33 | *gga-mir-6651* | 4.83 | *UHRF2* | 4.17 |
| *ZCCHC24* | 8.11 | *TUBB3* | 6.03 | *GNAS* | 5.33 | *RIT2* | 4.75 | *PPWD1* | 4.02 |
| *TBC1D5* | 7.17 | *DEF8* | 6.03 | *Edn3* | 5.23 | *WDR72* | 4.72 | *CZH5ORF44* | 4.02 |
| *TPGS2* | 6.42 | *DBNDD1* | 6.03 | *ARID1B* | 5.20 | *SLC35F3* | 4.67 | *TRIM23* | 4.02 |
| *KIAA1328* | 6.42 | *MC1R* | 6.03 | *CACNA2D1* | 5.17 | *ADK* | 4.54 | *ATXN2* | 3.98 |
| *CZH5ORF43* | 6.40 | *TCF25* | 6.03 | *DOCK4* | 5.06 | *gga-mir-6633* | 4.50 | *DTWD2* | 3.93 |
| *NDUFAF2* | 6.40 | *TUBB1* | 5.95 | *MYCT1* | 5.02 | *FANCA* | 4.33 | *JAKMIP2* | 3.92 |
| *NDUFS4* | 6.29 | *PTPN5* | 5.34 | *VIP* | 5.02 | *DNTT* | 4.28 | *NNT* | 3.88 |

**Figure S11** (A) Plots of the distributions of the log2(θπ ratio) and zFST values for comparisons between lowland and Tibetan chickens. (B) Selective sweep analysis of Tibetan chickens.

**Table S9** Functional gene categories enriched for genes under selection in the Tibetan chicken.

| Term | Term description | *P* value |
| --- | --- | --- |
| GO:0050877 | neurological system process | 9.14E-05 |
| GO:0006928 | cell motion | 2.68E-04 |
| GO:0051674 | localization of cell | 6.25E-04 |
| GO:0048870 | cell motility | 6.25E-04 |
| GO:0007169 | Transmembrane receptor protein tyrosine kinase signaling pathway | 0.00307 |
| GO:0007267 | cell-cell signaling | 0.0038666 |
| GO:0007268 | synaptic transmission | 0.003949 |
| GO:0016477 | cell migration | 0.0043132 |
| GO:0019226 | transmission of nerve impulse | 0.0073911 |
| GO:0050804 | regulation of synaptic transmission | 0.0085413 |
| GO:0042325 | regulation of phosphorylation | 0.0086924 |
| GO:0051969 | regulation of transmission of nerve impulse | 0.0093558 |
| GO:0031644 | regulation of neurological system process | 0.0093558 |
| GO:0051174 | regulation of phosphorus metabolic process | 0.0099851 |
| GO:0019220 | regulation of phosphate metabolic process | 0.0099851 |
| GO:0007166 | cell surface receptor linked signal transduction | 0.0112091 |
| GO:0007167 | enzyme linked receptor protein signaling pathway | 0.0119969 |
| GO:0044057 | regulation of system process | 0.0126515 |
| GO:0007611 | learning or memory | 0.0149262 |
| GO:0035258 | steroid hormone receptor binding | 0.018944 |
| GO:0005355 | glucose transmembrane transporter activity | 0.0251805 |
| GO:0050890 | cognition | 0.0361998 |
| GO:0015145 | monosaccharide transmembrane transporter activity | 0.0436586 |
| GO:0005154 | epidermal growth factor receptor binding | 0.0436586 |
| GO:0015149 | hexose transmembrane transporter activity | 0.0436586 |

Note: Only GO-BP, GO-MF, KEGG-pathway or InterPro domain terms with P values less than 0.05 are listed. P-values are corrected by the Benjamini-Hochberg FDR.

**A**

| Chr | SNP position | TC | LC | Mutation type | Annotation | Gene name |
| --- | --- | --- | --- | --- | --- | --- |
| chr1 | 119832182 | 0.90 | 0.17 | synonymous | ENSGALT00000026640:exon23:c.T2637T:p.D879D | *GPR64* |
| chr1 | 119856480 | 0.90 | 0.17 | synonymous | ENSGALT00000026654:exon18:c.A1873A:p.I625I | *PHKA2* |
| chr1 | 119862981 | 0.15 | 0.85 | synonymous | ENSGALT00000026654:exon25:c.G2706G:p.S902S | *PHKA2* |
| chr4 | 1578212 | 0.95 | 0.20 | synonymous | ENSGALT00000006334:exon3:c.T2746T:p.W916W | *BCORL1* |
| chr4 | 1610685 | 0.20 | 0.80 | nonsynonymous | ENSGALT00000006314:exon9:c.T1093C:p.S365P | *ZDHHC9* |
| chr8 | 13042656 | 0.89 | 0.15 | synonymous | ENSGALT00000009552:exon17:c.T1962C:p.S654S | *EVI5* |
| chr8 | 13071241 | 0.90 | 0.19 | nonsynonymous | ENSGALT00000009552:exon19:c.C2293A:p.Q765K | *EVI5* |
| chr10 | 19270887 | 0.19 | 0.85 | synonymous | ENSGALT00000013352:exon8:c.A996G:p.P332P | *CTDSPL2* |
| chr19 | 6666772 | 0.90 | 0.20 | synonymous | ENSGALT00000007405:exon5:c.T819T:p.F273F | *TRPV1* |
| chr6 | 17088763 | 0.88 | 0.13 | synonymous | ENSGALT00000009261:exon12:c.C1836C:p.D612D | *PKD2L1* |
| chr6 | 17090528 | 0.20 | 0.90 | nonsynonymous | ENSGALT00000009261:exon15:c.G2263A:p.E755K | *PKD2L1* |

**B**

**Figure S12** (A) SNPs in coding sequences with extreme differences in allele frequencies between Tibetan chickens and other domestic chickens. The annotation information is presented in the following format (separated by colons) Ensembl Transcript ID: Exon number: Reference allele + SNP position in the transcript + variant allele: Reference amino acid + codon position in the transcript + predicted amino acid change. Differences in allele frequency between two groups were analyzed using Pearson’s chi-squared test. The P values from Pearson’s chi-squared test were <0.001 for all alleles. (B) Mutations in the coding region of EVI5. The sequence is aligned with the orthologous protein sequences from 4 vertebrates, and the mutant residues are indicated by shading. The gene coordinate is based on Ensembl ID ENSGALG00000005935. The NJ tree derived from the multiple alignment (right panel) is shown in the left panel. ‘‘.’’ indicates the same amino acid, and ‘‘-’’ indicates an amino acid deletion.

**Table S10** Number of bins with SNP ≥20 in each window.

| **Bin size** | **Number of bins** | **Number of bins with SNP (≥20)** | **Ratio of bins with SNP (≥20)** |
| --- | --- | --- | --- |
| 1Mb | 1,962 | 1,536 | 0.783 |
| 500kb | 2,964 | 2,537 | 0.856 |
| 200kb | 5,974 | 5,539 | 0.927 |
| 100kb | 10,978 | 10,531 | 0.959 |
| 40kb | 25,946 | 25,356 | 0.977 |
| 20kb | 50,846 | 48,514 | 0.954 |
| 10kb | 100,233 | 86,994 | 0.868 |
